# Activation-gated, T cell-restricted silencing of *Dapk1* enhances Bacille Calmette-Guérin-elicited protective CD4^+^ memory

**DOI:** 10.1016/j.isci.2026.115593

**Published:** 2026-04-03

**Authors:** Zixuan Liu, Weihuang Liu, Chenyu Zhao, Xuanchang Bai, Fangting Zhou, Hang Sun, Jiahe Shi, Pinru Chen, Min Liu, Qin Pan

**Affiliations:** 1 Department of Anatomy, Hubei Province Key Laboratory of Allergy and Immunology, Wuhan University TaiKang Medical School (School of Basic Medical Sciences), Wuhan, China; 2 Medical Research Center for Structural Biology, Wuhan University TaiKang Medical School (School of Basic Medical Sciences), Wuhan, China; 3 Department of Immunology, Hubei Province Key Laboratory of Allergy and Immunology, Wuhan University Taikang Medical School (School of Basic Medical Sciences), Wuhan, China

**Keywords:** biological sciences, molecular biology, immunology, biotechnology

## Abstract

Durable CD4^+^ T cell memory is essential against mycobacterial infection, yet activation-induced cell death (AICD) limits the survival of activated clones after BCG vaccination. The upstream, cell-intrinsic brakes that govern this bottleneck remain incompletely defined. Combining transcriptomics, loss-of-function tests, and *in vivo* engineering, we identify *Dapk1* as a pro-apoptotic regulator that is downregulated in memory CD4^+^ T cells and promotes activation-induced death in T cell models. We develop an activation-gated AAV platform in which an NFAT-IL-2 promoter drives Cre to flip a FLEXed U6-shRNA cassette, and package them into a single AIO vector. This design confines *Dapk1* silencing to antigen-experienced T cells, preferentially within the CD4^+^CD44^hi^ compartment. In BCG-vaccinated mice, transient activation-linked *Dapk1* inhibition expands CD4^+^ T_CM_ cells, enhances IL-2 and Th1-skewed recall responses, lowers pulmonary, and splenic bacterial burdens after *M.tb* challenge. These findings highlight a strategy to selectively modulate intrinsic death pathways during immune priming for strengthening vaccine-elicited T cell memory.

## Highlights

• Downregulated *Dapk1* in memory CD4^+^ T cells promotes activation-induced death
• An activation-gated “all-in-one (AIO)” AAV silences *Dapk1 in vivo*
• AIO expands CD4^+^ T_CM_ and enhances Th1 recall responses in BCG-vaccinated mice
• Activation-dependent *Dapk1* silencing reduces *M.tb* burden in BCG-vaccinated mice

## Graphical abstract

**Figure:**
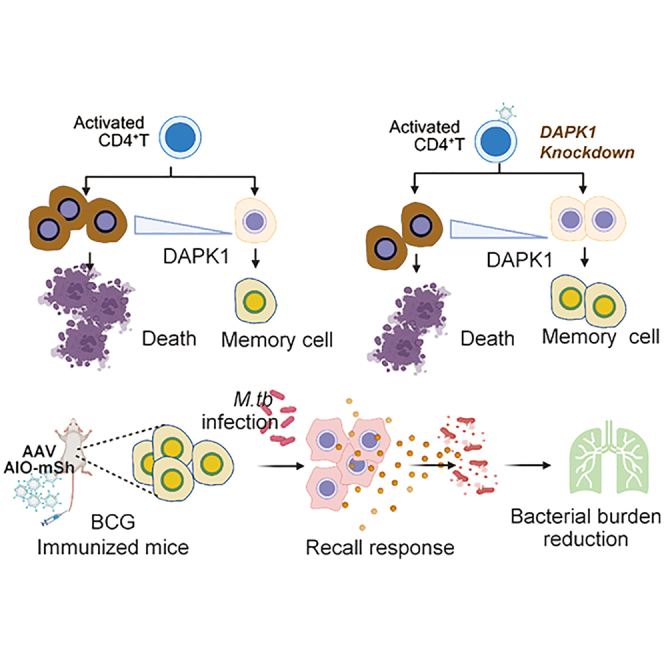

## Introduction

Tuberculosis (TB), caused by *M.tb*, remains a major global health challenge despite widespread use of the BCG vaccine, whose protective efficacy in adults is inconsistent.^1^^,^^2^^,^^3^ Following vaccination, naive T cells pass through activation, proliferation, a contraction phase, and the formation of memory T cells. Among these, CD4^+^ central memory T cell (T_CM_) cells are particularly important for durable protection.^4^^,^^5^ Effective antimycobacterial immunity further relies on CD4^+^ T helper 1 (Th1) cells capable of producing IFN-γ, and TNF-α, highlighting the importance of enhancing both the magnitude and quality of memory responses.^6^^,^^7^ However, how BCG drives T_CM_ and the regulatory mechanisms that limit or sustain this pool remain incompletely defined. Thus, exploring adjuvant vaccination strategies designed to strengthen TB vaccine-induced Th1 memory cells is a critical need.

Death-associated protein kinase-1 (DAPK1) is a calmodulin-dependent serine/threonine kinase with established roles in apoptosis, autophagy, and immune regulation.^8^^,^^9^ In T cells, DAPK1 influences proliferation, differentiation and antiviral function,^10^^,^^11^^,^^12^ but its impact on memory T cell formation has not been clarified. This gap suggests that DAPK1-mediated cell-intrinsic death pathways could be a modifiable barrier to the survival of activated clones that seed long-lived memory.

AAV vectors offer a practical route for *in vivo* gene modulation owing to low immunogenicity and broad tropism,^13^^,^^14^ yet efficient and selective targeting of lymphocytes, especially activated T cells, remains challenging. Wild-type AAV serotypes (e.g., AAV8) show predominant liver tropism after intravenous delivery and only limited infection of splenic T cells, which can confound immune-focused interventions.^14^^,^^15^ These limitations motivate strategies that gate gene regulation to activated T cells rather than relying solely on serotype tropism.

Here, we set out to test whether transient silencing of *Dapk1* in activated T cells enhances BCG-elicited CD4^+^ memory and early control of mycobacterial infection. We first delivered AAV2/8-sh*Dapk1* systemically after BCG vaccination to assess effects on memory formation and recall, and then engineered a Cre-dependent FLEXed U6-shRNA cassette driven by an NFAT-IL-2 promoter to confine knockdown to activated T cells. Finally, we combined these elements into a single “AIO” AAV for one-virus delivery and validated its efficiency. Across these platforms we evaluated memory subset composition, cytokine recall, bacterial burden after *M. tb* challenge, and lung histopathology. In brief, the activation-gated approach selectively reduced *Dapk1* in CD4^+^CD44^hi^ cells, expanded the T_CM_ pool with IL-2 dominant recall, and lowered pulmonary and splenic colony-forming units following the challenge.

## Results

### Memory phenotype CD4^+^ T cells downregulate *DAPK1*

**Figure 1:**
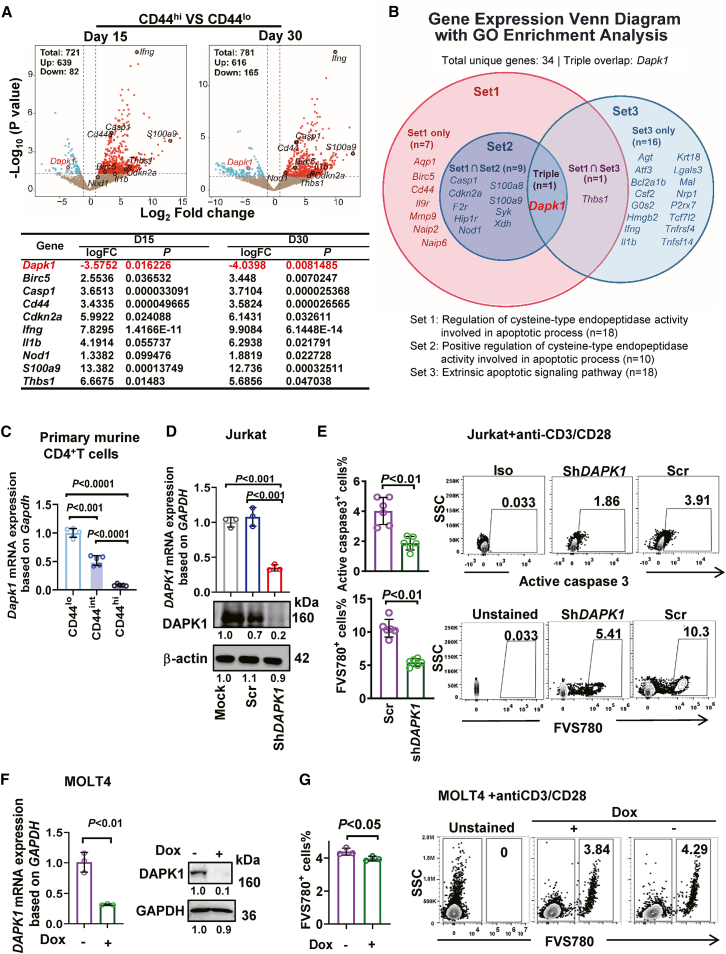
Memory phenotype CD4^+^ T cells downregulate *DAPK1* (A and B) *Dapk1* is transcriptionally downregulated in CD44^hi^CD4^+^ T cells and converges on apoptotic pathways. (A) Volcano plot displaying DEGs (CD44^hi^ vs. CD44^lo^). Key apoptosis-related genes are labeled with log_2_FC and *p* values. (B) Venn diagram illustrating that *Dapk1* is uniquely shared across three apoptotic pathways. (C) *Dapk1* mRNA expression was quantified by RT-qPCR in CD44^lo^, CD44^int^, and CD44^hi^CD4^+^ T cell fractions and normalized to *Gapdh* (*n* = 5). (D) Validation of DAPK1 knockdown by RT-qPCR (upper side) and immunoblotting (lower side) in Jurkat cells stably expressing sh*DAPK1* or a scramble control (*n* = 3). (E) FCM quantification of intracellular active caspase-3 (upper side) and cell viability (using FVS780 dye, lower side) in Jurkat cells 24 h after activation with anti-CD3/CD28 antibodies (*n* = 6). (F) Doxycycline-inducible knockdown of DAPK1. DAPK1 mRNA and protein levels were assessed by RT-qPCR and immunoblotting, respectively, in MOLT-4 cells stably expressing a Dox-inducible sh*DAPK1* construct 48 h after Dox treatment (*n* = 3). (G) FCM quantification of cell viability in the same MOLT-4 cells as in (F) (*n* = 3). Data are presented as the mean ± SD. Comparisons between two groups were analyzed using unpaired two-tailed Student’s *t* test. Comparisons among three or more groups were analyzed using one-way ANOVA followed by Tukey’s post hoc test.

To investigate factors influencing CD4^+^ T cell immune memory, we subcutaneously injected WT mice with BCG. CD44^lo^ and CD44^hi^ populations within splenic CD4^+^ T cells were sorted by flow cytometry (FCM) on day 15 and 30 post-BCG vaccination for bulk RNA-seq analysis. We comprehensively analyzed all differentially expressed genes (DEGs), including both upregulated and downregulated genes (Figure 1A). Given our study’s focus on cell death and stress responses, we selected twenty-two GO pathways closely related to these themes from the enrichment results for further investigation (Figures 1A and S1A). This analysis revealed *Dapk1* as a consistently downregulated gene that uniquely mapped to three pivotal apoptotic pathways: (1) regulation of cysteine-type endopeptidase activity, (2) positive regulation of cysteine-type endopeptidase activity, and (3) extrinsic apoptotic signaling (Figures 1B and S1B). Its position across these pathways marked *Dapk1* as a putative hub gene in the apoptosis network.

RT-qPCR across CD44^lo/int/hi^ fractions confirmed a stepwise decline in *Dapk1* mRNA with increasing CD44 expression, indicating that downregulation accompanies acquisition of the memory program (Figure 1C). Functionally, stable shRNA knockdown in Jurkat T cells lowered the frequency of active caspase-3^+^ cells after TCR-mimetic stimulation and reduced viability dye defined death (Figures 1D and 1E). In MOLT-4 T cells, a doxycycline (Dox)-inducible shRNA decreased both DAPK1 protein and transcript upon Dox induction (Figure 1F) and, after anti-CD3/CD28 stimulation, yielded fewer viability dye positive cells than uninduced controls (Figure 1G). Together, these data show that memory like CD4^+^ T cells are characterized by reduced *DAPK1*, and that lowering *DAPK1* dampens apoptotic execution following T cell activation.

### Systemic AAV-sh*Dapk1* broadens CD4^+^ memory and augments cytokine recall

**Figure 2:**
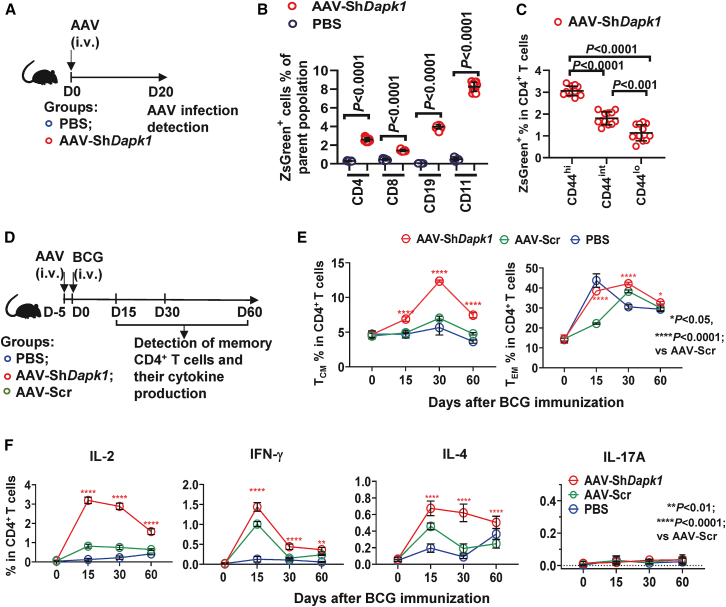
Systemic AAV-sh*Dapk1* broadens CD4^+^ memory and augments cytokine recall (A–C) AAV2/8-ZsGreen-sh*Dapk1* achieves reporter ZsGreen expression across splenic lineages. (A) Schematic diagram. Mice were intravenously injected with AAV2/8-ZsGreen-sh*Dapk1* on day 0, and ZsGreen expression were measured by FCM on day 20. (B) ZsGreen expression in splenic CD4^+^ T, CD8^+^ T, CD19^+^ B and CD11b^+^ myeloid cells (*n* = 12). (C) ZsGreen expression in splenic CD44^hi^CD4^+^, CD44^int^CD4^+^ and CD44^lo^CD4^+^ T cells (*n* = 12). (D–F) Analysis of memory and cytokine-producing CD4^+^ T cells following BCG immunization. (D) Schematic diagram. On day −5, mice were intravenously injected with AAV2/8-ZsGreen-sh*Dapk1*. On day 0, these mice were intravenously immunized with BCG. FCM was performed on the indicated days. (E) Longitudinal frequencies of T_CM_ and T_EM_ CD4^+^ T cells (*n* = 6). (F) Cytokine production following *ex vivo* stimulation with inactivated BCG. The frequencies of IL-2^+^, IFN-γ^+^, IL-4^+^, and IL-17A^+^ cells among CD4^+^ T cells were determined (*n* = 4). Data are presented as mean ± SD. Statistical significance was assessed using one-way ANOVA followed by Tukey’s post hoc test.

Having established that *DAPK1* promotes activation-induced death and is downregulated in memory phenotype CD4^+^ T cells, we next tested whether transient *in vivo* silencing of *Dapk1* during BCG priming would broaden the CD4^+^ memory compartment and enhance antigen-specific recall responses (Figure 2A). Following intravenous delivery, AAV2/8-ZsGreen-sh*Dapk1* drove reporter (ZsGreen) expression in multiple splenic cell lineages, including CD4^+^ T cells (Figures 2B, S2, and S3A–S3C). Within the CD4^+^ T cell compartment, reporter signal was notably enriched in CD44^hi^ subsets (Figure 2C). Longitudinal profiling from day 15 through day 60 post-BCG revealed a sustained increase in CD4^+^ T_CM_ cells (CD44^hi^CD62L^hi^) in sh*Dapk1*-treated mice compared with PBS and scramble controls, with divergence evident by day 15 and maintained or amplified at later time points; effector memory CD4^+^ T cells (T_EM_; CD44^hi^CD62L^lo^) were likewise elevated over the same interval, indicating that *Dapk*1 silencing during priming supports both the reservoir of high-proliferative potential cells and their effector-biased counterparts (Figures 2D, 2E, and S3D).

Functional recall assays mirrored these compositional changes. Upon *in vitro* restimulation with inactivated BCG, sh*Dapk1* markedly increased IL-2 production and also elevated IFN-γ and TNF-α relative to controls (Figures 2F and S3E–S3H). The shift toward a stronger IL-2/Th1-skewed recall response is consistent with the expanded T_CM_ pool and suggests improved proliferative competence and helper function. Our results demonstrated that temporary *Dapk1* silencing during the priming/expansion phase confers a sustained advantage to memory formation.

Because systemic AAV delivery led to detectable reporter expression in multiple splenic lineages, we next sought to develop an activation-gated, CD4^+^ T-cell-targeted AAV vector to restrict *Dapk1* silencing to activated CD4^+^ T cells.

### A Cre dependent FLEXed U6-shRNA cassette enables conditional *Dapk1* silencing

**Figure 3:**
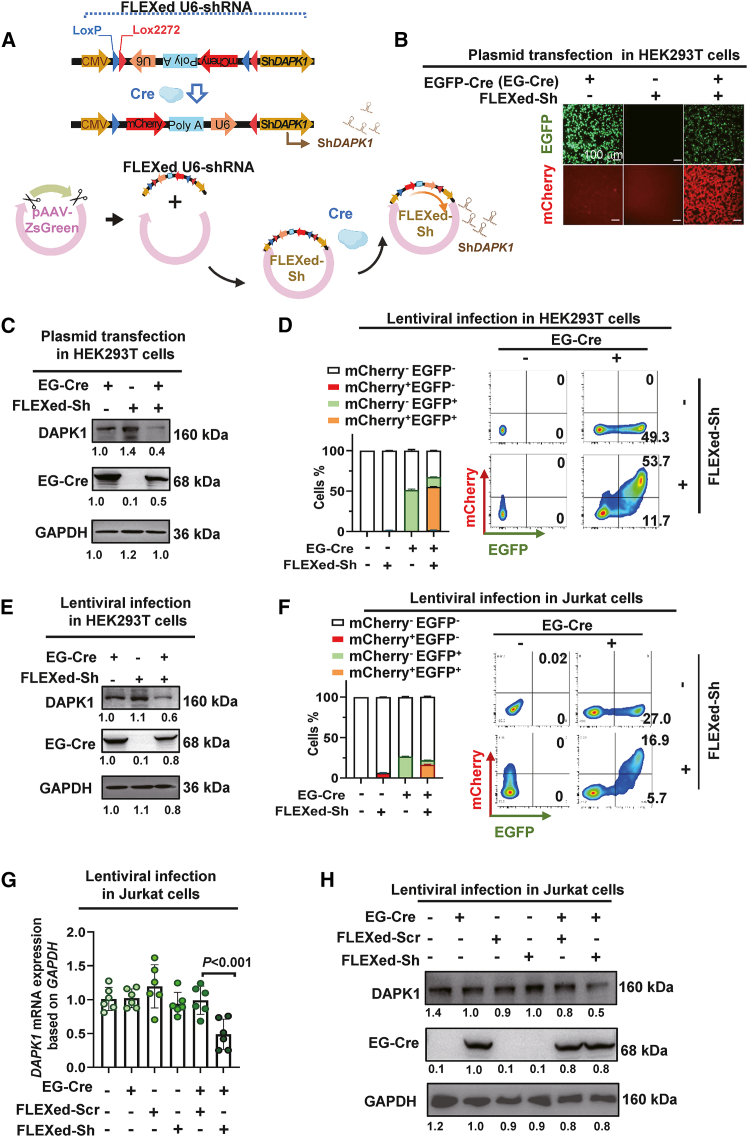
A Cre-dependent FLEXed U6-shRNA cassette enables conditional *Dapk1* silencing (A) Schematic of the FLEXed U6-shRNA cassette. The cassette contains a U6 promoter and polyA signal that are flanked by heterologous *loxP* and lox2272 sites and placed in an inverse (transcriptionally silent) orientation. Upon Cre-mediated recombination, the promoter/polyA unit is inverted into the correct orientation, allowing transcription of the downstream sh*DAPK1* sequence. This genetic switch is coupled to a constitutive CMV-driven mCherry reporter, which provides a direct fluorescent readout for successful recombination. (B and C) HEK293T cells were co-transfected with FLEXed U6-sh*DAPK1* and EGFP-Cre (EG-Cre) plasmids. The inversion of the shRNA cassette was analyzed by fluorescence microscopy and immunoblotting 48 h post-transfection. (B) Analysis of the EGFP^+^/mCherry^+^ double-positive cell population by fluorescence microscopy. Scale bar, 100 μm. (C) Immunoblotting analysis of DAPK1 from the cell lysates. (D and E) Lentiviral delivery of Cre triggers inversion of the FLEXed shRNA cassette in HEK293T cells. Cells were transduced with lentivirus encoding the FLEXed U6-sh*DAPK1* construct and EG-Cre. Inversion of the cassette and *DAPK1* silencing were analyzed 60 h post-transduction by FCM and immunoblotting. (D) FCM quantification of double-positive cells. Left side, pooled data (*n* = 4). Right side, representative flow plots. (E) Immunoblotting analysis of DAPK1 from the cell lysates. (F–H) Lentiviral delivery of Cre triggers inversion of the FLEXed shRNA cassette in Jurkat cells. The cells were transduced with the lentivirus as described for (D). (F) FCM quantification of double-positive cells. Left side, pooled data (*n* = 4). Right side, representative flow plots. (G) RT-qPCR validation of *DAPK1* mRNA levels (*n* = 6). (H) Immunoblotting analysis of DAPK1 protein levels. Data are presented as the mean ± SD. Statistical significance was assessed using one-way ANOVA followed by Tukey’s post hoc test.

To confine *DAPK1* knockdown to defined cellular contexts, we engineered a double-floxed inverse orientation (DIO) construct. This design places a U6-poly A motif flanked by *loxP*/lox2272 sites in an inverse orientation, and is inverted into the correct transcriptional orientation upon Cre-mediated recombination. sh*DAPK1* is followed by the motif to construct FLEXed U6-shRNA cassette. The construct is coupled with an CMV-driven mCherry reporter that is activated in parallel, providing a direct fluorescent readout of successful recombination (Figure 3A).

In HEK293T cotransfection experiments, introduction of Cre together with the DIO construct generated a clear EGFP^+^/mCherry^+^ double-positive population only when the FLEXed cassette was present, indicating successful inversion and reporter activation under Cre control (Figure 3B). Immunoblotting of matched lysates showed a reduction of DAPK1 protein that occurred specifically when Cre and sh*DAPK1* were combined, whereas Cre alone and or sh*DAPK1* alone did not alter DAPK1 abundance (Figure 3C). This result was recapitulated in a viral delivery system. Following lentiviral transduction of HEK293T cells, Cre expression successfully triggered mCherry activation from the FLEXed cassette (Figures 3D and 3E). Flow cytometric quantification confirmed consistent increases in EGFP^+^/mCherry^+^ double-positive cells relative to controls (Figure 3D). Furthermore, immunoblotting demonstrated a corresponding reduction of DAPK1 in the cells transduced with Cre and sh*DAPK1* (Figure 3E). Extending this to T cells, Jurkat cultures receiving Cre together with sh*DAPK1* exhibited mCherry reporter expression alongside a drop in DAPK1 protein, demonstrating that the system operates in the intended lineage (Figures 3F–3H), and population-level analysis in Jurkat corroborated Cre-dependent inversion and target suppression with negligible effects in scramble or Cre-negative groups (Figures 3F–3H). Across formats, FLEXed U6-shRNA inversion and *DAPK1* knockdown tracked together at the single-cell level, validating the reporter as a faithful readout of functional cassette flipping. These data establish a modular platform for conditional *DAPK1* silencing that can be layered onto activation- or lineage-specific Cre sources.

### An NFAT-IL-2 promoter drives Cre to confine FLEXed U6-shRNA inversion to activated T cells

**Figure 4:**
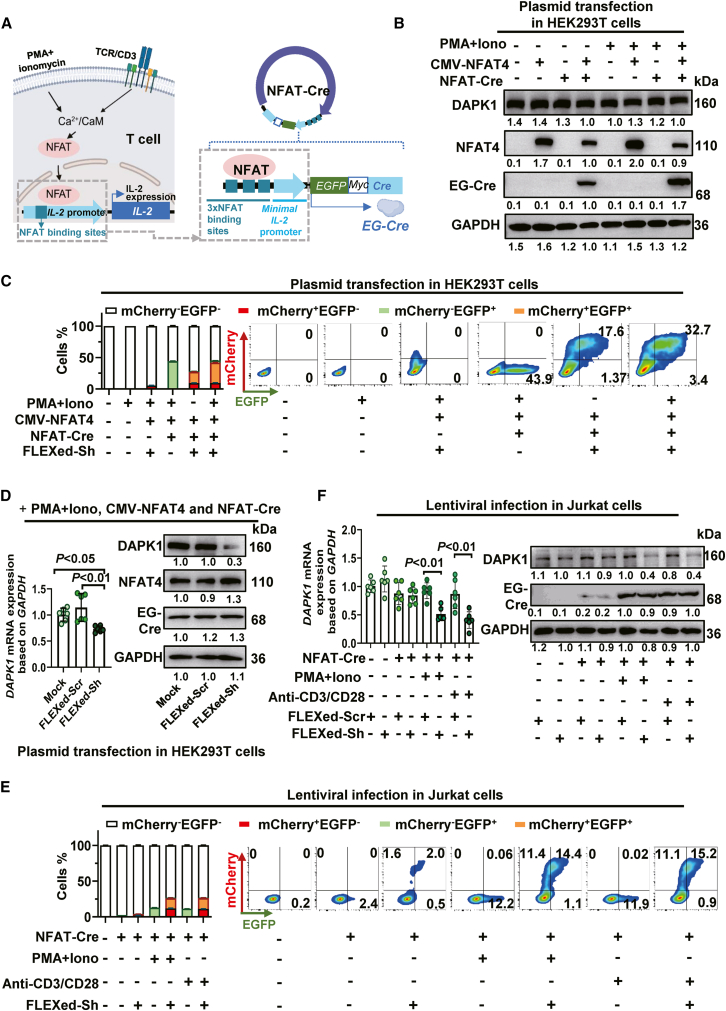
An NFAT-IL-2 promoter drives Cre to confine FLEXed U6-shRNA inversion to activated T cells (A) Design of NFAT-Cre. A synthetic 3×NFAT-minimal IL-2 promoter drives expression of EG-Cre. Upon TCR signaling, calcium flux induces NFAT translocation to the nucleus, which triggers EG-Cre expression. This design ensures that inversion of the FLEXed cassette occurs only upon T cell activation. (B) Immunoblotting validated that PMA/ionomycin treatment and overexpression of NFAT4 in the cells had no effect on DAPK1 expression. (C and D) HEK293T cells were co-transfected with FLEXed U6-sh*DAPK1*, NFAT-Cre, and CMV-NFAT4 plasmids. The inversion of the shRNA cassette was analyzed by FCM, RT-qPCR, and immunoblotting 48 h post-transfection. (C) FCM quantification of double-positive cells. Left side, pooled data (*n* = 6). Right side, representative flow plots. (D) Left side, RT-qPCR validation of *DAPK1* mRNA levels (*n* = 6). Right side, immunoblotting analysis of DAPK1 protein levels. (E and F) Lentiviral delivery of NFAT triggers inversion of the FLEXed shRNA cassette in Jurkat cells upon activation. The cells were transduced with the lentivirus encoding the FLEXed U6-sh*DAPK1* construct and NFAT-Cre. After 60 h of transduction, the cells were activated with anti-CD3/CD28 for an additional 12 h. The inversion of the cassette and *DAPK1* silencing were analyzed. (E) FCM quantification of double-positive cells. Left side, pooled data (*n* = 6). Right side, representative flow plots. (F) Analysis of DAPK1 expression. Right side, RT-qPCR validation of *DAPK1* mRNA levels (*n* = 6). Right side, immunoblotting analysis of DAPK1 protein levels. Data are presented as the mean ± SD. Statistical significance was assessed using one-way ANOVA followed by Tukey’s post hoc test.

Then we engineered a T cell activation-dependent system for silencing *DAPK1*. Based on the principle that TCR signaling activates NFAT via calcium flux, we placed Cre under the control of a synthetic 3×NFAT-minimal IL-2 promoter (Figure 4A). This design ensures that inversion of the FLEXed cassette occurs only upon T cell activation and NFAT translocation. Immunoblotting analysis showed that transfection with NFAT-Cre and treatment with PMA/ionomycin (which mimics T cell receptor activation) had no effect on DAPK1 protein levels in HEK293T cells (Figure 4B). However, cotransfection of NFAT-Cre and FLEXed cassette confirmed that the NFAT-gated genetic switch was functional, as activation markedly increased the proportion of EGFP^+^/mCherry^+^ double-positive cells, while resting cultures showed a low basal signal, indicating tight NFAT control (Figure 4C). We next assessed the functional output of the NFAT-gated knockdown system in the double-positive population. Cells treated with sh*DAPK1* exhibited selective reductions in DAPK1 mRNA and protein, while scramble controls showed no effect (Figure 4D). This confirms that the NFAT-dependent knockdown functions precisely in the intended lineage.

Extending these findings to T cells, Jurkat cells were transduced with lentivirus encoding NFAT-Cre and a FLEXed U6-shRNA cassette and then stimulated via anti-CD3/CD28 or PMA/ionomycin. As expected, the cells exhibited an increased EGFP^+^/mCherry^+^ double-positive population and reduced mRNA and protein levels of DAPK1 in sh*DAPK1*-treated cells (Figures 4E and 4F). These results show that NFAT-Cre gates the inversion of the FLEXed U6-shRNA cassette and thus *DAPK1* silencing specifically to activated T cells.

### A single AIO construct enables single-virus, activation-dependent *DAPK1* silencing in T cells

**Figure 5:**
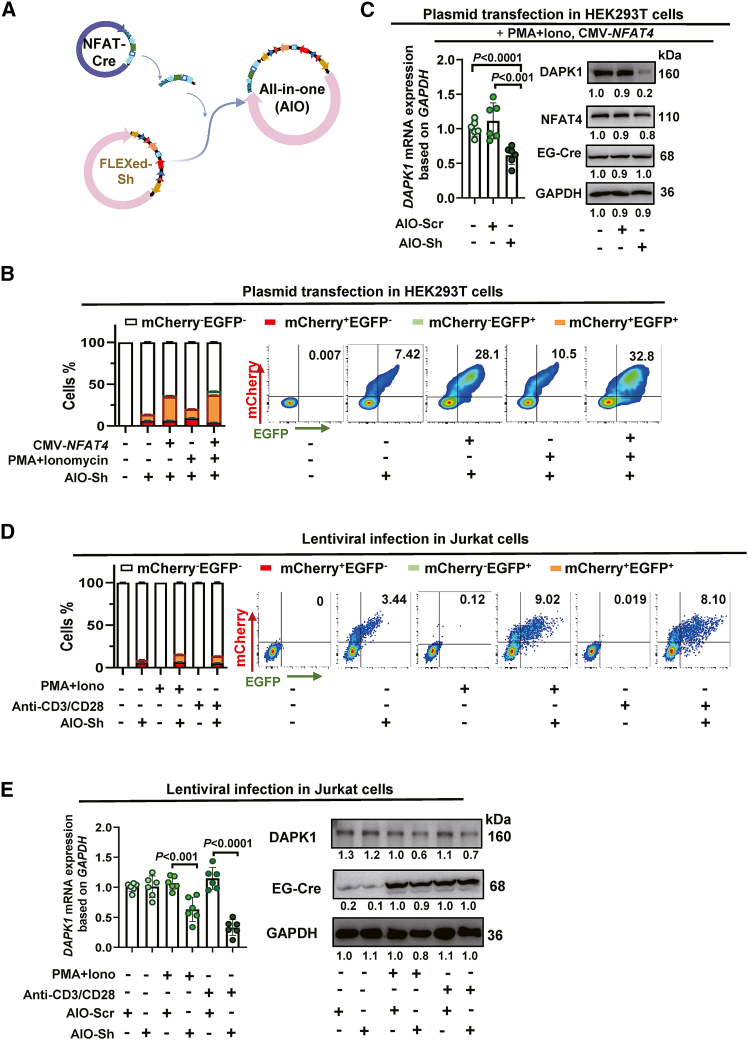
AIO enables single-virus, activation-dependent DAPK1 silencing (A) Schematic of the AIO construct combining FLEXed U6-sh*DAPK1* and NFAT-Cre. (B and C) HEK293T cells were transfected with AIO plasmid. The inversion of the shRNA cassette was analyzed by FCM, RT-qPCR and immunoblotting 48 h post-transfection. (B) FCM quantification of double-positive cells. Left side, pooled data (*n* = 5). Right side, representative flow plots. (C) Left side, RT-qPCR validation of *DAPK1* mRNA levels (*n* = 6). Right side, immunoblotting analysis of DAPK1 protein levels. (D and E) Lentiviral delivery of activation-dependent *DAPK1* silencing in Jurkat cells. Cells were transduced with lentivirus encoding the AIO construct. At 60 h post-transduction, cells were activated with anti-CD3/CD28 for an additional 12 h. Cassette inversion and *DAPK1* silencing were then analyzed. (D) FCM quantification of double-positive cells. Left side, pooled data (*n* = 6). Right side, representative flow plot. (E) Analysis of DAPK1 expression. Right side, RT-qPCR validation of *DAPK1* mRNA levels (*n* = 6). Right side, immunoblotting analysis of DAPK1 protein levels. Data are presented as the mean ± SD. Statistical significance was assessed using one-way ANOVA followed by Tukey’s post hoc test.

To simplify delivery while preserving activation gating, we combined NFAT-Cre, a FLEXed U6-shRNA cassette and dual reporters on a single AAV genome to develop an AIO construct (Figure 5A). In NFAT over-expressing HEK293T cells, PMA/ionomycin activation increased the fraction of EGFP^+^/mCherry^+^ double-positive cells, indicating that the AIO retains tight NFAT dependence (Figure 5B). Molecular readouts confirmed function, after activation, AIO-sh*DAPK1* but not AIO-scramble, showed Cre expression together with a decrease in *DAPK1* transcript and protein, demonstrating on-target silencing within the single-vector format (Figure 5C). In anti-CD3/CD28 activated Jurkat cells, the same pattern held, the AIO-sh*DAPK1* group showed an increased EGFP^+^/mCherry^+^ double-positive population and reduced DAPK1 mRNA and protein relative to the AIO-scramble control (Figures 5D and 5E). These results suggest that the single-vector architecture preserves the activation gate while simplifying production and transduction. This is a key feature for systemic administration *in vivo*, where constraints on multiplicity of infection and co-transduction often limit multi-vector strategies.

### The AIO system mediates preferential knockdown of *Dapk1* transcripts in activated CD4^+^ T cells in mice

**Figure 6:**
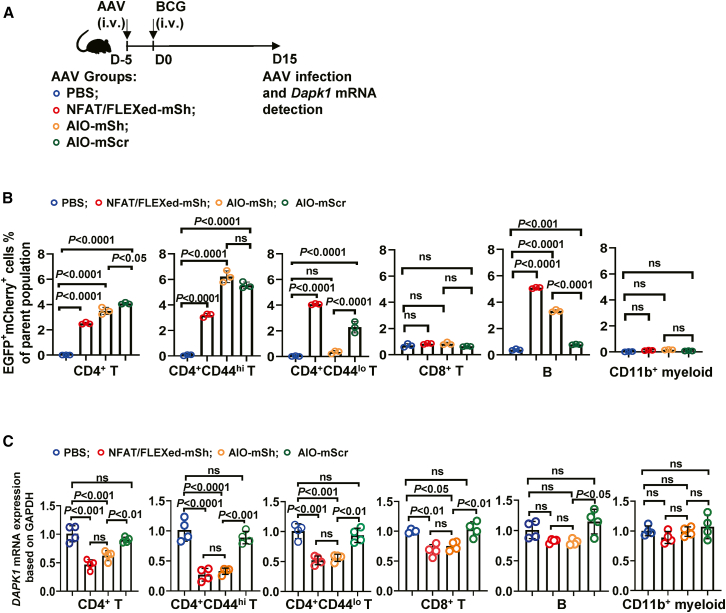
The AIO system mediates preferential *Dapk1* knockdown in activated CD4^+^ T cells *in vivo* (A–C) Mice received AAVs encoding a *Dapk1*-targeting shRNA under the control of either the AIO system (AIO-mSh) or an NFAT-Cre/FLEXed system (NFAT/FLEXed-mSh) 5 days before BCG immunization. (A) Schematic diagram. (B) FCM analysis showing EGFP^+^/mCherry^+^ double-positive cells, indicating shRNA expression, in various splenic cell populations (*n* = 3). (C) RT-qPCR confirmation of *Dapk1* mRNA knockdown in the sorted cell subsets (*n* = 4). Data are presented as the mean ± SD. Statistical significance was assessed using one-way ANOVA followed by Tukey’s post hoc test.

We assessed the effects of AIO-sh*DAPK1* on memory T cell development using a murine model of BCG vaccination. The mice were intravenously (*i.v.*) injected with AAV encoding AIO-mouse-sh*Dapk1* (AIO-mSh) or NFAT-Cre/FLEXed U6-mouse-sh*Dapk1* (NFAT/FLEXed-mSh) on day −5, and vaccinated (*i.v.*) with BCG on day 0. The efficacy of murine *Dapk1* RNA silencing was then assessed on day 15 (Figure 6A). After systemic AAV delivery, the dual-reporter readout (EGFP and mCherry) revealed that recombination events were concentrated within the CD4^+^ T cell and B cell compartments and were markedly enriched among CD44^hi^ relative to CD44^lo^ CD4^+^ T cells. A small but significant increase in dual-reporter-positive CD11b^+^ myeloid cells was also detected; however, their low frequency (<0.2%) indicates weak knockdown efficacy in this cell type (Figure 6B). Subset-resolved RT-qPCR demonstrated a selective reduction of *Dapk1* mRNA in sorted CD4^+^CD44^hi^ T cells from NFAT-Cre/FLEXed U6-mshRNA and AIO-msh RNA groups. Consistent with the reporter activity profile (Figure 6B), a modest but significant knockdown of *Dapk1* was detected in CD4^+^CD44^lo^ T cells, CD8^+^ T cells and B cells, reflecting off-target shRNA activity outside the primary CD4^+^CD44^hi^ target compartment. Myeloid cells showed little to no change (Figures 6C and S4). Moreover, *Dapk1* mRNA levels in scramble or PBS controls were indistinguishable from baseline in CD4^+^ T cells (Figures 6C and S4). Together, these results confirm that the activation-gated AIO strategy operates as designed *in vivo*, favoring CD4^+^CD44^hi^ T cells and achieving targeted suppression of *Dapk1* with minimal impact on non-T cell lineages.

### Activation-dependent *Dapk1* silencing enhances BCG-elicited memory and improves early control of *M.tb*

**Figure 7:**
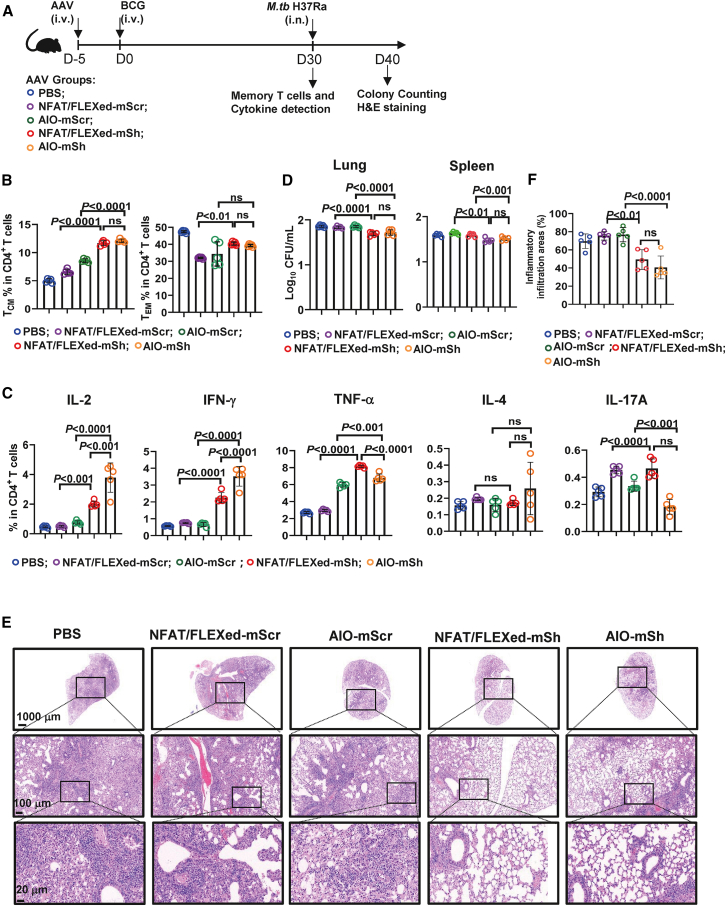
Activation-dependent *Dapk1* silencing enhances BCG-elicited memory and improves early control of *M.tb* (A–E) Mice were administered AAVs encoding a *Dapk1*-targeting shRNA under the control of either AIO-mSh or NFAT/FLEXed-mSh 5 days before BCG immunization. Intranasal infection with *M.tb* H37Ra was performed 30 days post-immunization. (A) Schematic diagram. (B) FCM analysis of memory CD4^+^ T cells 30 days after BCG immunization (*n* = 5). (C) FCM analysis of cytokine-producing CD4^+^ T cells after *ex vivo* restimulation with BCG (*n* = 5). (D) Lung and spleen bacterial burden following *M.tb* challenge (*n* = 5). (E and F) Histopathological assessment of lung tissue after challenge. (E) Representative hematoxylin and eosin (H&E)-stained lung tissue sections. Scale bars, 1000 μm (top), 100 μm (middle), and 20 μm (bottom). (F) Quantification of inflammatory infiltration area in lung sections, expressed as a percentage of total tissue area (*n* = 5). Data are presented as the mean ± SD. Statistical significance was assessed using one-way ANOVA followed by Tukey’s post hoc test.

To determine the impact of T cell restricted *Dapk1* silencing on protective immunity, we challenged AAV-injected, BCG-vaccinated mice with *M.tb* H37Ra at day 30. We then measured T cell memory, recall function, bacterial burden, and lung pathology (Figure 7A). By day 30 post the vaccination, the mice receiving NFAT-Cre/FLEXed U6-mshRNA and AIO-mshRNA exhibited higher frequencies of CD4^+^ T_CM_ cells than PBS or scramble controls, with CD4^+^ T_EM_ cells showing a similar upward trend, indicating that activation-gated *Dapk1* knockdown during priming expands memory compartments (Figures 7B, S5A, and S5B). Interestingly, these *Dapk1* knockdown mice showed a slight increase in CD8^+^ T_CM_ cells and a marked increase in CD8^+^ T_EM_ cells (Figures S5C–S5F). Upon *ex vivo* restimulation with inactivated BCG, the *Dapk1* knockdown mice showed a Th1-biased CD4^+^ T cell response, characterized by increased frequencies of IL-2 producing cells and elevated levels of IFN-γ and TNF-α, but minimal changes in IL-4 and IL-17A (Figures 7C and S5B). Furthermore, CD8^+^ T cells in these mice exhibited increased production of granzyme B, IFN-γ, and TNF-α (Figures S5C–S5F).

Building on this BCG-specific recall response to shared mycobacterial antigens, we next evaluated protective immunity using an attenuated *M. tb* H37Ra challenge model. Upon intranasal challenge, pulmonary and splenic colony-forming units were reduced in the *Dapk1*-silenced groups relative to controls, demonstrating improved early bacterial control (Figure 7D). Histopathology of lung tissue revealed fewer and smaller inflammatory foci and better-preserved alveolar architecture in NFAT-Cre/FLEXed U6-mshRNA and AIO-mshRNA groups, indicating mitigation of tissue damage alongside reduced pathogen burden (Figures 7E and 7F). Collectively, confining *Dapk1* knockdown to activated T cells strengthens BCG-driven memory formation and confers measurable early control of mycobacterial infection.

## Discussion

This study identifies DAPK1 as a cell-intrinsic regulator that limits the survival of activated CD4^+^ T cells and constrains the establishment of BCG-elicited memory. We show that *Dapk1* is transcriptionally reduced in memory phenotype CD4^+^ T cells, that loss of DAPK1 decreases activation-induced apoptosis in T cell models, and that transient silencing of *Dapk1* during priming expands the CD4^+^ T_CM_ compartment and strengthens IL-2 dominant recall responses. By engineering an activation-gated AAV platform, placing Cre under an NFAT-IL-2 promoter to flip a FLEXed U6-shRNA cassette, we confine *Dapk1* knockdown to antigen-experienced T cells *in vivo*. This design enhances BCG-driven memory and improves early control of *M.tb* after challenge.

The combined transcriptional and functional data support a model in which DAPK1 promotes AICD downstream of TCR signaling. Reduced DAPK1 in CD44^hi^CD4^+^ T cells is consistent with a survival advantage in the cells that progress into memory. In cell lines, DAPK1 loss diminished active caspase-3 and viability dye defined death after stimulation, indicating that DAPK1 participates in apoptotic execution. *In vivo*, *Dapk1* silencing increased the frequency of CD4^+^ T_CM_ and elevated IL-2 and IFN-γ during recall. IL-2 is tightly linked to memory precursors with high proliferative potential, and its increase aligns with the expansion of T_CM_. Together, these findings suggest that transient attenuation of DAPK1 at the time of activation allows more clones to survive contraction and enter the memory pool without grossly perturbing resting cells.

To assess the functional impact of the enhanced memory pool, we employed an attenuated *M. tb* challenge model. Compared to the scrambled (Scr) control, active *Dapk1* knockdown (shRNA groups) not only reduced bacterial burden (Figure 7D) but also significantly ameliorated lung histopathology (Figure 7F). This demonstrates that *Dapk1* suppression enhances protective immunity, improving both bacterial control and tissue-level outcomes. The modest absolute difference in CFU likely reflects the compressed dynamic range of bacterial clearance in this short-term, attenuated model, whereas the pronounced histological improvement underscores a direct benefit on immunopathology.

Having established the efficacy of *Dapk1* suppression, we compared the two *Dapk1* knockdown constructs. Both constructs achieved comparable core efficacy, but with a notable difference in their TNF-α signature (Figures 7C–7F). The optimized, single-vector AIO system uniquely resulted in a lower frequency of TNFα-producing CD4^+^ T cells (Figure 7C). This distinct AIO signature may be an indicator of more coordinated, and potentially more metabolically or resource-efficient, cellular regulation. The AIO design ensures that activation-gating and gene knockdown are tightly coupled within the same cell. This may allow T cells to achieve the necessary functional output with a more restrained inflammatory profile, avoiding the full “cost” of a broader cytokine storm. It suggests that the AIO platform can achieve robust immunity while potentially sparing T cell resources, a feature that might be advantageous for durable responses or in hosts with limited immune reserves.

The ability to achieve such specific immunomodulation highlights the importance of our engineered delivery system. A major challenge in modulating lymphocyte pathways *in vivo* is achieving sufficient specificity to avoid bystander effects. Systemic AAV8 targeting alone showed broad tropism,^14^^,^^15^^,^^16^ whereas the NFAT-IL-2 promoter provided a functional gate that is naturally engaged during T cell activation. The DIO architecture adds a second layer of control: only when Cre is expressed does the U6 promoter flip to drive shRNA, simultaneously reporting inversion by mCherry. *In vivo*, reporter inversion and *Dapk1* transcript reduction were most prominent in CD4^+^CD44^hi^ cells. Packaging Cre and FLEXed U6-shRNA into a single AIO vector simplifies dosing and co-transduction requirements, a practical consideration for translation beyond proof-of-principle studies.

According to our *in vivo* results, while *Dapk1* knockdown is most significant in CD4^+^CD44^hi^ T cells, a milder, yet statistically significant reduction is also observed in CD4^+^CD44^lo^ and CD8^+^ T cells, indicating off-target activity in these compartments. However, the knockdown efficiency in CD4^+^CD44^hi^ cells is greater than in CD44^lo^ or CD8^+^ T cells (Figure 6C), making it the most likely driver of the observed phenotypic changes. Moreover, the enhanced memory formation and cytokine recall (Figure 7) are quantitatively correlated with the extent of *Dapk1* reduction specifically within the CD4^+^CD44^hi^ compartment. The functional outcomes (expanded T_CM_, enhanced IL-2) are hallmarks of CD4^+^ T cell helper function, making this lineage the most parsimonious source of the observed effect.

Interestingly, we also observed an expansion of CD8^+^ T_EM_ cells following *Dapk1* silencing (Figure S5C right panel). This finding appears at odds with reports that DAPK1 promotes mTORC1 activation and that its genetic deletion can impair CD8^+^ T cell activation and trafficking.^11^^,^^12^ We propose that this apparent discrepancy can be reconciled through a two-tiered mechanism. First, a direct cell-intrinsic effect may occur. Our system achieves partial, transient *Dapk1* knockdown (not complete knockout) primarily in antigen-experienced CD44^hi^CD8^+^ T cells. This partial reduction may be insufficient to cause a strong activation defect but could subtly shift the balance away from AICD toward memory precursor survival during the recall response. Second, the CD8^+^ T_EM_ expansion is best explained by an indirect, CD4^+^ T cell-mediated extrinsic effect. Our NFAT-gated intervention is designed to target CD4^+^ T cells. The consequent enhancement of CD4^+^ T_CM_ survival and function creates an altered helper microenvironment. The augmented provision of cytokines such as IL-2 and IFN-γ from these robust helpers can overcome any potential mild intrinsic deficit in CD8^+^ T cells and powerfully drive their expansion and T_EM_ differentiation. This “helper-override” model aligns with the well-established paradigm that CD4^+^ T cell help dictates the magnitude and quality of CD8^+^ T cell memory.^17^^,^^18^^,^^19^

BCG efficacy varies widely in adults, in part because vaccine-elicited memory can be suboptimal. Our data indicate that removing a death-promoting brake in activated T cells improves both the size and quality of the memory CD4^+^ compartment. The strengthened IL-2/Th1-biased recall response is immunologically meaningful in mycobacterial control and was accompanied by lower bacterial burdens and improved lung histopathology after challenge. Conceptually, this work frames transient, activation-restricted gene modulation as a “genetic adjuvant” that complements classical pathogen- or pattern receptor-based adjuvants. Because the gate is driven by a transcriptional program engaged by many vaccines, the platform could, in principle, be repurposed to other antigens and T cell intrinsic targets.

Broad inhibition of apoptosis can carry risks of lymphoproliferation or autoimmunity.^20^^,^^21^^,^^22^ Several features of the design mitigate these concerns. First, *Dapk1* silencing is activation-dependent; resting lymphocytes lack Cre and thus do not express the shRNA. Second, the intervention is transient: AAV-driven shRNA expression decays over time as episomes dilute in proliferating cells, and inversion occurs only in cells that receive sufficient NFAT signaling. Third, our *in vivo* phenotyping did not reveal gross perturbations in non-T compartments within the sampling window. Nonetheless, long-term monitoring will be important to exclude delayed effects, particularly after repeated vaccination or exposure to unrelated antigens.

NFAT-responsive promoters can be active in other leukocytes under certain stimuli.^23^^,^^24^^,^^25^ We also detected some reporter activity in B cells of BCG-vaccinated mice. Promoter refinements, such as combining NFAT with T cell restricted elements or microRNA target de-targeting, could further tighten specificity. DIO leakiness was low in our model, but future iterations could incorporate optimized lox spacing, ribozymes, or degrons to minimize basal expression.

By coupling a vaccine-induced transcriptional gate to a Cre-switchable shRNA, we selectively inhibited a pro-apoptotic kinase in activated T cells and improved CD4^+^ memory and early control of mycobacterial infection. The results indicate DAPK1 as a promising target for preserving antigen-experienced clones and demonstrate that activation-gated, T cell-restricted gene modulation can augment vaccine efficacy while minimizing effects on resting or bystander cells. This platform provides a flexible foundation for next generation immunomodulatory adjuvants aimed at durable, high quality T cell immunity.

### Limitations of the study

There are some limitations in the study. While the intranasal *M.tb* H37Ra challenge model was suitable for our initial proof-of-concept, evaluating the full adjuvant potential of AIO-mediated *Dapk1* silencing will require challenge with virulent *M. tb* H37Rv via the standard aerosol route. Our findings are based on intravenous BCG vaccination. Future studies should include mucosal vaccination routes to identify the optimal BCG administration strategy for this approach. Moreover, the 30-day vaccination-to-challenge interval and 10-day endpoint post challenge were selected to specifically interrogate the early phase of recall-mediated bacterial control, which aligns with our primary mechanistic focus on the initial impact of *Dapk1* silencing. Longer vaccination-to-challenge intervals (≥60–90 days) and later post-challenge endpoints (≥21–30 days) will be required to evaluate memory maintenance, tissue remodeling, and durable protection to further confirm the impact on CD4^+^ T_CM_ cells. The study focused on CD4^+^ T cells; effects on CD8^+^ or unconventional T cells were not deeply characterized and merit evaluation, as DAPK1 may differentially regulate survival across lineages. We did not directly measure TCR repertoire breadth, transcriptional states of memory precursors, or long-term durability beyond the early memory window. Single cell transcriptomic and epigenomic profiling could define whether *Dapk1* silencing selectively preserves stem like memory subsets and how it intersects with pathways governing metabolic fitness, autophagy, and stress responses. Finally, AAV immunity and pre-existing neutralizing antibodies can affect vector performance in humans, engineered capsids or non-viral delivery may be required for clinical translation.

## Resource availability

### Lead contact

• Requests for further information and resources should be directed to and will be fulfilled by the lead contact, Qin Pan (panqincn@whu.edu.cn).

### Materials availability

• All plasmids, reagents and cell lines mouse lines generated in this study are available from the lead contact.

### Data and code availability

• RNA-seq have been deposited at the CNCB Genome Sequence Archive (GSA; China National Center for Bioinformation) under CNCB Genome Sequence Archive: CRA011545. The deposited data are publicly available from the date of publication.
• This manuscript did not generate new code.
• Any additional information required to reanalyze the data reported in this paper is available from the lead contact upon request.

## Acknowledgments

This work was supported by 10.13039/501100012166National Key R&D Program of China (2022YFA1303500), the 10.13039/501100001809National Natural Science Foundation of China (grant nos. 32570221, 82230078, and 82272978).

## Author contributions

Conceptualization, Q.P. and M.L.; experimental setup and development, Z.L., W.L., C.Z., and X.B.; AAV preparation, F.Z.; immunoblotting, H.S.; murine models, J.S.; analysis, Q.P. and P.C.; software, F.Z. and J.S.; and writing and editing, Q.P. and M.L.

## Declaration of interests

The authors declare no competing interests.

## Declaration of generative AI and AI-assisted technologies in the writing process

The authors did not use generative AI or AI-assisted technologies in the preparation of this manuscript.

## STAR★Methods

### Key resources table

**Table:** 

REAGENT or RESOURCE	SOURCE	IDENTIFIER
**Antibodies**		

Anti-mouse CD3		
Anti-human CD3	Biolegend	UCHT1; Cat# 300438; RRID: AB_11146991
Anti-human CD28	Biolegend	CD28.2; Cat# 302934; RRID: AB_11148949
Anti-mouse CD28	Biolegend	37.51; Cat# 102116; RRID: AB_11147170
Anti-caspase-3	BD Biosciences	C92-605.rMAb;Cat# 570184; RRID: AB_3685573
Anti-GAPDH	Proteintech	1E6D9; Cat# 60004-1-Ig; RRID: AB_2107436
Anti-Myc	ABclonal	AMC0504; Cat# AE010; RRID: AB_2770408
Anti-Flag	MBL International	FLA-1; Cat# M185-3L; RRID: AB_11123930
Anti-DAPK1	Signalway Antibody	Cat# 33007;RRID: AB_3716584
PE/Cyanine7 anti-mouse CD3	Biolegend	17A2; Cat# 100220; RRID: AB_1732057
Brilliant Violet 650(TM) anti-mouse CD3	Biolegend	17A2; Cat# 100229; RRID: AB_11204249
Brilliant Violet 605(TM) anti-mouse CD8a	Biolegend	53-6.7; Cat# 100744; RRID: AB_2562609
Alexa Fluor(R) 700 anti-mouse CD4	Biolegend	RM4-5;Cat#100536;RRID: AB_493701
Brilliant Violet 650™ anti-mouse TNF-α	Biolegend	MP6-XT22; Cat# 506333; RRID: AB_2562450
PE anti-mouse IL-17A	Biolegend	TC11-18H10.1; Cat# 506928; RRID: AB_2629787
Alexa Fluor® 647 anti- mouse IL-4	Biolegend	11B11; Cat# 504112; RRID: AB_493323
Brilliant Violet 421™ anti-mouse IL-2	Biolegend	JES6-5H4; Cat# 503826; RRID: AB_2650897
PE anti-mouse IFN-γ	Biolegend	XMG1.2; Cat# 505808; RRID: AB_315402
PE anti-human/mouse Granzyme B	Biolegend	QA18A28; Cat# 396405; RRID: AB_2801074
Brilliant Violet 421(TM) anti-mouse/human CD44	Biolegend	IM7; Cat# 103040;; RRID: AB_2616903

**Bacterial and virus strains**		

*M.tb* H37Rv	CMCC	93009
Bacillus Calmette-Guérin (BCG)	ATCC	35734

**Chemicals, peptides, and recombinant proteins**		

PMA	Solarbio	Cat#P6714;RRID: AB_3716591
Ionomycin	Solarbio	Cat#II2200;RRID: AB_3716592
Ultra sensitive ECL chemiluminescence substrate	Biosharp	Cat# BL523B
Zombie NIRTM Fixable Viability Kit	Biolegend	Cat# 423105
Fixation Buffer	Biolegend	Cat# 420801
Intracellular Staining Perm Wash Buffer(10×)	Biolegend	Cat# 421002
Brefeldin A	Absin	Cat# abs810012
Cell lysis buffer	Beyotime	Cat# P0013
Fixable Viability Stain 780 (FVS780)	BD Biosciences	Cat#565388

**Critical commercial assays**		

CD4^+^ T Cell Isolation Kit	Miltenyi Biotec	Cat# 130-104-454
Amicon® Ultra, 100 kDa MWCO	MilliporeSigma	Cat# UFC910096
Polyvinylidene difluoride membrane	Millipore	Cat# IPVH00010

**Deposited data**		

Bulk RNA-Seq	This paper	Genome Sequence Archive (GSA, CRA accession: CRA011545)

**Experimental models: Cell lines**		

Human: HEK293T	ATCC	Cat# CRL-3216
Jurket cells MOLT-4	ATCC ATCC	Cat# TIB-152 Cat# CRL-1582

**Oligonucleotides**		

Oligonucleotides for genes Knockdown		
*NFAT4*-F: 5’ACAAGCTTGCGGCCGCGATGA CTACTGCAAACTGTGGCG3’	This paper	N/A
*NFAT4*-R: 5’ACAGGGATGCCACCCGGTTAG AGCCCATCAGATCTTCCTA3’ DAPK1 (human) -F 5’CCGGCCACGTCGATACCTTG AAATTTCAAGAGAATTTCAAGG TATCGACGTGGTTTTTG3’	This paper	N/A
*DAPK1* (human) -R 5’AATTCAAAAACCACGTCGAT ACCTTGAAATTCTCTTGAAATT TCAAGGTATCGACGTGG3’	This paper	N/A
*Dapk1* (mouse) -F 5’GATCGCTTGATATCACTGTG CCAAACTCGAGTTTGGCACA GTGATATCAAGCTTTTTG3’	This paper	N/A
*Dapk1* (mouse) -R 5’AGCTCAAAAAGCTTGATATC ACTGTGCCAAACTCGAGTTTG GCACAGTGATATCAAGC3’	This paper	N/A
Primers for RT-qPCR		
Primer: DAPK1 (human) -F 5’ GAGGAACTTGGCAGTGGACA3’	This paper	N/A
Primer: DAPK1 (human) -R 5’ CATAGACCTCGTGCAGGGTG3’	This paper	N/A
Primer: Dapk1 (mouse) -F 5’ AGATGTGGTCCGCTACCTCTGT3’	This paper	N/A
Primer: Dapk1 (mouse) -R 5’ ATGCTCGTGCTGTTCTGCCTTG3’	This paper	N/A
Primer: *ITR*-F 5’ GGAACCCCTAGTGATGGAGTT3’	This paper	N/A
Primer: *ITR*-R 5’ CGGCCTCAGTGAGCGA3’	This paper	N/A
Primer: *GAPDH* (human) -F 5’ GTCTCCTCTGACTTCAACAGCG3’	This paper	N/A
Primer: *GAPDH* (human) -R 5’ ACCACCCTGTTGCTGTAGCCAA3’	This paper	N/A
Primer: *Gapdh* (mouse) -F 5’ CATCACTGCCACCCAGAAGACTG3’	This paper	N/A
Primer: *Gapdh* (mouse) -R 5’ ATGCCAGTGAGCTTCCCGTTCAG3’	This paper	N/A
Gene synthesis		
FLEXed-U6		
5’ACCGCGTCATTAGTTCATAGGGGCAGAG CGCACATCGCCCACAGTCCCCGAGAAG TTGGGGGGAGGGGTCGGCAATTGAACG GGTGCCTAGAGAAGGTGGCGCGGGGTA AACTGGGAAAGTGATGTCGTGTACTGG CTCCGCCTTTTTCCCGAGGGTGGGGGA GAACCGTATATAAGTGCAGTAGTCGCC GTGAACGTTCTTTTTCGCAACGGGTTT GCCGCCAGAACACAGGGACGGTACCC TTCAGAGCTCGGTCGATAACTTCGTATA GCATACATTATACGAAGTTATCCGAATC GCAATAACTTCGTATAGGATACTTTATA CGAAGTTATACTCTCGAGGTCCTTTCCA CAAGATATATAAAGCCAAGAAATCGAAA TACTTTCAAGTTACGGTAAGCATATGA TAGTCCATTTTAAAACATAATTTTAAAA CTGCAAACTACCCAAGAAATTATTACTT TCTACGTCACGTATTTTGTACTAATATC TTTGTGTTTACAGTCAAATTAATTCCAA TTATCTCTCTAACAGCCTTGTATCGTAT ATGCAAATATGAAGGAATCATGGGAAA TAGGCCCTCTCTAGAACCATAGAGCCCA CCGCATCCCCAGCATGCCTGCTATTGTC TTCCCAATCCTCCCCCTTGCTGTCCTGC CCCACCCCACCCCCCAGAATAGAATGA CACCTACTCAGACAATGCGATGCAATTT CCTCATTTTATTAGGAAAGGACAGTGG GAGTGGCACCTTCCAGGGTCAAGGAA GGCACGGGGGAGGGGCAAACAACAG ATGGCTGGCAACTAGAAGGCACAGAG CTCGCTGATCAGCCTCGATCCTAAACC TACTTGTACAGCTCGTCCATGCCGCCG GTGGAGTGGCGGCCCTCGGCGCGTTC GTACTGTTCCACGATGGTGTAGTCCT CGTTGTGGGAGGTGATGTCCAACTTG ATGTTGACGTTGTAGGCGCCGGGCAGC TGCACGGGCTTCTTGGCCTTGTAGGTG GTCTTGACCTCAGCGTCGTAGTGGCCG CCGTCCTTCAGCTTCAGCCTCTGCTTG ATCTCGCCCTTCAGGGCGCCGTCCTCG GGGTACATCCGCTCGGAGGAGGCCTCC CAGCCCATGGTCTTCTTCTGCATTACG GGGCCGTCGGAGGGGAAGTTGGTGCC GCGCAGCTTCACCTTGTAGATGAACTC GCCGTCCTGCAGGGAGGAGTCCTGGGT CACGGTCACCACGCCGCCGTCCTCGAA GTTCATCACGCGCTCCCACTTGAAGCCC TCGGGGAAGGACAGCTTCAAGTAGTCG GGGATGTCGGCGGGGTGCTTCACGTAG GCCTTGGAGCCGTACATGAACTGAGGG GACAGGATGTCCCAGGCGAAGGGCAGG GGGCCACCCTTGGTCACCTTCAGCTTGG CGGTCTGGGTGCCCTCGTAGGGGCGGC CCTCGCCCTCGCCCTCGATCTCGAACTC GTGGCCGTTCACGGAGCCCTCCATGTGC ACCTTGAAGCGCATGAACTCCTTGATGAT GGCCATGTTATCCTCCTCGCCCTTGCTCA CCATGGTGGCGCTAGCCGTAATAGTATA ACTTCGTATAATGTATGCTATACGAAGTT ATCCGAATCGCAATAACTTCGTATAAAGT ATCCTATACGAAGTTATACGGTAACCTGA CCGGTCCACGTCGATACCTTGAAATTTCA AGAGAATTTCAAGGTATCGACGTGGTTT TTGAATTCAGTAAGCTT3’	This paper	N/A
NFAT-IL2 promoter 5’ACTAGTGGAGGAAAAACTGTTT CATACAGAAGGCGTACGCCTTC TGTATGAAACAGTTTTTCCTCCA CGCCTTCTGTATGAAACAGTTTT TCCTCCTCGAGGACATTTTGACA CCCCCATAATATTTTTCCAGAATT AACAGTATAAATTGCATCTCTTGT TCAAGAGTTCCCTATCACTCTCTT TAATCACTACTCACAGTAACCTCA ACTCCTGCGAATTC3’	This paper	N/A

**Deposited Data**		

Bulk RNA-Seq	This paper	CNCB Genome Sequence Archive: CRA011545

**Software and algorithms**		

Prism V9 or later	GraphPad Software	https://www.graphpad.com/
Flowjo V10.6.2	BD Biosciences	https://www.flowjo.com/
Image J Version 1.54j	NIH	https://imagej.net/software/imagej/
R studio version 4.2.0	R Foundation for Statistical Computing	https://www.r-project.org/

### Experimental model and study participant details

#### Microbe strains

*Mycobacterium bovis* BCG (ATCC 93009) and *Mycobacterium tuberculosis* H37Ra (ATCC 25177) were maintained on Lowenstein–Jensen medium and subcultured into Middlebrook 7H9 broth (oleic acid albumin dextrose catalase, 0.05% Tween-80) for liquid growth. Aliquots were prepared at log phase and stored at −80 °C. Prior to use, suspensions were vortexed with sterile glass beads and briefly sonicated (water bath) to disperse clumps; CFU were verified by serial dilution on 7H10 agar.

#### Cell lines

HEK293T cells were cultured in DMEM and Jurkat and MOLT-4 T cells in RPMI-1640; all media contained 10% FBS and 1× penicillin/streptomycin. Cells were maintained at 37 °C in a humidified 5% CO_2_ incubator, routinely tested mycoplasma-negative, and passaged at 70-80% confluence. For activation, Jurkat/MOLT-4 cells were stimulated with anti-CD3 (2 μg mL^-1^) plus anti-CD28 (2 μg mL^-1^) or PMA (50 ng mL^-1^) plus ionomycin (1 μM) for the indicated times.

#### Mice

Female C57BL/6J wild-type mice (6-8 weeks) were bred under specific-pathogen-free conditions at the Animal Laboratory Center of Wuhan University. Animals were housed under a 12-hour light/dark cycle with *ad libitum* access to food and water. All procedures were approved by the Institutional Animal Care and Use Committee of Wuhan University (No. 2017077 and No. 2017095). Work involving live mycobacteria was conducted in an Animal Biosafety Level-3 (ABSL-3) facility with institutional biosafety approval.

### Method details

#### Plasmid design and cloning

FLEXed U6-shRNA cassette. To enable Cre-dependent silencing, a double-floxed inverse orientation (DIO) module was built with heterotypic loxP/lox2272 sites flanking an inverted U6 promoter, inverted bovine growth hormone (bGH) poly(A). An mCherry reporter was included within the same DIO block, ensuring it flips in parallel with the U6 promoter to provide a single-cell readout of recombination. The shRNA sequence (sh*DAPK1* for human cell lines or sh*Dapk1* for mice) is followed by the downstream loxP/lox2272 site. A scramble control was constructed identically with a non-targeting hairpin. Sequences and oligonucleotides are listed in the key resources table.

NFAT-Cre. To restrict recombination to activated T cells, Cre-EGFP (myc-tagged) was placed under a synthetic promoter comprising three tandem NFAT sites upstream of the IL-2 minimal promoter.

AIO construct. For single-virus delivery, NFAT-Cre and FLEXed U6-shRNA cassette were combined on a single AAV genome (ITR-bounded), maintaining total size within packaging limits. AIO-scramble was constructed identically with a non-targeting hairpin.

EGFP-Cre. Cre recombinase was expressed in HEK293T and Jurkat cells using plasmid and lentiviral constructs in which an EGFP-tagged Cre is driven by a CMV promoter.

CMV-NFAT4. A CMV-NFAT4 plasmid was used to overexpress NFAT4 in HEK293T. NFAT4 was placed under a CMV promoter.

All constructs were sequence-verified. Plasmids were propagated in Stbl3 bacteria and purified with endotoxin-free kits.

#### AAV production and titration

Recombinant AAV2/8 was produced by triple transfection of HEK293T cells in 15-cm dishes using polyethylenimine (PEI, 1 mg mL^-1^ stock; 1:3 DNA: PEI mass ratio). For each dish, 20 μg transfer plasmid, 20 μg AAV2 rep/AAV8 cap plasmid, and 40 μg adenoviral helper plasmid were mixed in Opti-MEM. After a 20 min incubation, PEI was added dropwise. After 72 hours of culture, the cell lysates and supernatants were collected together. The mixture was treated with chloroform (0.1 volume), vortexed, and shaken vigorously at 37 °C for 1 h. Solid NaCl was added to a final concentration of 1 mol/L, followed by centrifugation at 3000 g for 5 min at 4 °C. The aqueous phase was collected, supplemented with PEG8000 to 10% (w/v), vortexed, and incubated on ice for additional 1 hour. After centrifugation (3000 g, 30 min, 4 °C), the pellets were resuspended in PBS (0.5 mL per original 15 cm dish) and pooled. The intermediate AAV product was supplemented with MgCl_2_ to 2 mM and Benzonase to 250 U/mL, then digested at 37 °C for 30 min. The digest was aliquoted, mixed with an equal volume of chloroform, vortexed, and centrifuged (3000 g, 5 min, 4 °C). Chloroform extraction was repeated 2-3 times until the aqueous phase was clear. The final aqueous phase contained purified AAV, which was aliquoted and stored at -80 °C. Genomic titers (gc mL^-1^) were determined by qPCR against ITR or EGFP sequences using a linearized plasmid standard curve. Endotoxin levels were confirmed to be <5 EU mL^-1^.

#### HEK293T cell co-transfection

Cells were co-transfected with FLEXed U6-shRNA and NFAT-Cre, or transfected with AIO plasmid. At 48 hours, reporter inversion was quantified by FCM (EGFP^+^/mCherry^+^ double-positive frequency), and DAPK1 protein was assessed by immunoblotting in RIPA lysates (30 μg protein/lane).

#### Jurkat/MOLT-4 assays

Cells were transduced with lentiviral NFAT/FLEXed-Sh or AIO. After 60 hours of transduction, the cells were stimulated with anti-CD3/CD28 or PMA/ionomycin for an additional 12 h. DAPK1 mRNA was measured by RT-qPCR (ΔΔCt vs GAPDH), and DAPK1 protein was analyzed by immunoblotting. Apoptosis was analyzed by immunoblotting for active caspase-3 and by flow cytometry (FCM) using a fixable viability dye (FVS780). For doxycycline-inducible shRNA experiments, cells were treated with doxycycline (1 μg mL^-1^, 48-72 hours) prior to stimulation.

#### Murine model

Mice received a tail-vein injection of 1×10^12^ gc AAV2/8 in 100 μL PBS. Five days later, they were vaccinated intravenously (i.v.) with 1×10^6^ CFU BCG in 100 μL PBS. Thirty days post-vaccination, the mice were lightly anesthetized with isoflurane and challenged intranasally with 1×10^7^ CFU *M. tb* H37Ra in 50 μL PBS (25 μL per nostril). Animals were monitored daily, with predefined humane endpoints. To assess *Dapk1* silencing, tissues were harvested 20 days after AAV administration. To measure the memory T cells, splenocytes were prepared from the mice 30 days after vaccination for FCM analysis. To validate the recall protective response to *M. tb* H37Ra, lung and spleen tissues were collected 10 days after the challenge. Group sizes and exact time points are provided in the corresponding figure legends.

#### Tissue processing and cell preparation

Spleens were mechanically dissociated in cold RPMI followed by ACK red blood cell lysis. Cells were filtered, counted with trypan blue, and kept on ice prior to staining or culture.

#### FCM

Cell suspensions from murine spleens were stained with the fluorescent-conjugated primary antibodies. Standard flow cytometry staining was performed to detect: CD3, CD4, CD8, CD19, CD11b, CD44, CD62L. Memory subsets were defined as naïve (CD44^lo^CD62L^hi^), TCM (CD44^hi^CD62L^hi^), and TEM (CD44^hi^CD62L^lo^). Reporter expression (ZsGreen or EGFP/mCherry) was recorded to quantify AAV transduction and FLEXed cassette inversion. For intracellular staining, brefeldin A was added 6 hours before harvesting the cells. The cells were fixed/permeabilized with Cytofix/Cytoperm Fixation and Permeabilization Solution before incubation with anti-BMPG or anti-cytokine antibodies. Cells were detected using an FACSAria™ III flow cytometer and CytoFLEX flow cytometerat the Medical Structural Biology Research Center of the Wuhan University. FCM data were analyzed using FlowJo software. Detailed antibody information is listed in the key resources table.

#### Cell sorting and transcript analysis

CD4^+^ T, CD8^+^ T, CD19^+^ B, and CD11b^+^ myeloid cells were sorted to ≥95% purity on a FACSAria II. Total RNA was extracted with TRIzol, DNase-treated, and reverse-transcribed (random hexamers). qPCR was performed with SYBR Green chemistry on a 96-well platform. Relative *Dapk1* expression was calculated by the ΔΔCt method using *Gapdh* as internal control. Primer sequences are listed in the key resources table.

#### Bulk RNA-seq

Mice were primed (s.c.) with BCG (2 samples/group). The splenic CD3^+^CD4^+^CD44^hi^ and CD3^+^CD4^+^CD44^lo^ cells were sorted by FCM and used for RNA-seq. RNA integrity was evaluated by using the Agilent 2100 Bioanalyzer (Agilent Technologies, Santa Clara, CA, USA). RNA-seq libraries were constructed sequenced using BGISEQ 500. Clean reads were aligned to the reference genome and the reference genome using Bowtie2 v2.2.6 and HISAT v2.0.4. Gene expression level was represented by expected number of Fragments Per Kilobase of transcript sequence per Millions base pairs sequenced (FPKM), which was calculated by using RESM (RNA-Seq by Expectation-Maximization) software v1.2.12. Differential expression genes (DEGs) were considered significant according to P-value <0.05 and the absolute value of the log2(fold change) >1.

#### Immunoblotting

Cells were lysed in RIPA buffer with protease/phosphatase inhibitors. Protein concentration was measured by BCA assay. Equal amounts (20-40 μg) were resolved by SDS–PAGE, transferred to PVDF membranes, blocked (5% non-fat milk), and probed with antibodies against DAPK1, active caspase-3, and GAPDH (loading control). HRP-conjugated secondary antibodies and chemiluminescent substrate were used for detection; band intensities were quantified in ImageJ.

#### Bacterial burden (CFU)

Lungs and spleens were weighed separately and homogenized in sterile phosphate-buffered saline (PBS) with 0.05% Tween-80 (1 g tissue in 5 mL PBS), and serially diluted. Aliquots (100 μL) were plated in triplicate on Middlebrook 7H10 agar supplemented with OADC and incubated at 37 °C. Colonies were counted at 14-21 days. CFU were expressed as log_10_ (CFU per organ). Plates with 30-300 colonies were used for quantification; if all dilutions exceeded this range, the closest countable dilution was used and flagged.

#### Histology

Lung lobes were fixed in 4% paraformaldehyde (24-48 hous), paraffin-embedded, sectioned at 4-5 μm, deparaffinized, and stained with hematoxylin and eosin. Slides were scanned on a digital slide scanner; lesion area and inflammatory foci were quantified in blinded fashion using ImageJ with a predefined macro.

#### Randomization, blinding, and exclusion criteria

Mice were randomized to treatment groups immediately prior to AAV administration. Investigators were blinded to group allocation during CFU counting and histological scoring; FCM analysis was performed using standardized gate templates. Animals were excluded only if prespecified criteria were met (e.g., failed tail-vein injection evidenced by subcutaneous bleb, unexpected intercurrent illness unrelated to procedures). All final n are reported in figure legends.

### Quantification and statistical analysis

Data are presented as mean ± SD. Two-group comparisons used unpaired two-tailed Student’s t-test. Multi-group comparisons used one-way ANOVA with Tukey’s post-hoc test. Exact *n*, statistical tests, and *P* values are stated in the figure legends. Analyses were performed in GraphPad Prism (v9 or later); significance threshold was set at *P* < 0.05.

## References

[1] Singh U.B. (2025). BCG revaccination to prevent tuberculosis. N. Engl. J. Med..

[2] Carpenter S.M., Boom W.H. (2025). To BCG or not two BCG. N. Engl. J. Med..

[3] Danchuk S.N., Behr M.A. (2020). Bacille Calmette-Guérin: One hundred years, one hundred questions. Clin. Infect. Dis..

[4] Sterle H.M., Putz E.J., Olsen S.C., Boggiatto P.M. (2024). Induction of CD4 T-cell memory responses following BCG vaccination in cattle. Front. Vet. Sci..

[5] Dwivedi V., Gautam S., Headley C.A., Piergallini T., Torrelles J.B., Turner J. (2022). IL-10 receptor blockade delivered simultaneously with Bacillus Calmette-Guérin vaccination sustains long-term protection against Mycobacterium tuberculosis infection in mice. J. Immunol..

[6] Lindenstrøm T., Moguche A., Damborg M., Agger E.M., Urdahl K., Andersen P. (2018). T cells primed by live mycobacteria versus a tuberculosis subunit vaccine exhibit distinct functional properties. EBioMedicine.

[7] Rakshit S., Ahmed A., Adiga V., Sundararaj B.K., Sahoo P.N., Kenneth J., D'Souza G., Bonam W., Johnson C., Franken K.L. (2019). BCG revaccination boosts adaptive polyfunctional Th1/Th17 and innate effectors in IGRA+ and IGRA− adults. JCI Insight.

[8] Shiloh R., Bialik S., Kimchi A. (2014). The DAPK family: a structure–function analysis. Apoptosis.

[9] Zou S., Zhang C., Xu H., Liu Z., Hu Y., Wang W., Liu K., Wen Q., Song L. (2023). ISG20L1 acts as a co-activator of DAPK1 in the activation of the p53-dependent cell death pathway. J. Cell Sci..

[10] Yan M., Yao J., Lin Y., Yan J., Xie Y., Fu Z., Zhou Y., Wei J., Li X. (2023). Tumor cell density dependent IL-8 secretion induces the fluctuation of tregs/CD8+ T cells infiltration in hepatocellular carcinoma: one prompt for the existence of density checkpoint. J. Transl. Med..

[11] Wei Z., Du Q., Li P., Liu H., Xia M., Chen Y., Bi G., Tang Z.H., Cheng X., Lu Y. (2021). Death-associated protein kinase 1 (DAPK1) controls CD8+ T cell activation, trafficking, and antitumor activity. FASEB J..

[12] Wei Z., Li P., He R., Liu H., Liu N., Xia Y., Bi G., Du Q., Xia M., Pei L. (2021). DAPK1 (death associated protein kinase 1) mediates mTORC1 activation and antiviral activities in CD8+ T cells. Cell. Mol. Immunol..

[13] Pajaziti B., Michgehl U., Blazevic D., Fimm-Todt F., Duetting A., Korbmacher B., Grote-Wessels S., Michelfelder S., Mecklenburg L. (2026). Preclinical Assessment of Antibody Responses to Adeno-Associated Virus (AAV) Vector-Based Capsids of AAV2, AAV5, AAV8, or AAV9 in Laboratory Cynomolgus Macaques (*Macaca fascicularis*) of Asian or Mauritian Origin. Hum. Gene Ther..

[14] Moldavskii D., Gilazieva Z., Fattakhova A., Solovyeva V., Issa S., Sufianov A., Sufianova G., Rizvanov A. (2025). AAV-based gene therapy: opportunities, risks, and scale-up strategies. Int. J. Mol. Sci..

[15] Pierce G.F., Fong S., Long B.R., Kaczmarek R. (2024). Deciphering conundrums of adeno-associated virus liver-directed gene therapy: focus on hemophilia. J. Thromb. Haemost..

[16] Walkey C.J., Snow K.J., Bulcha J., Cox A.R., Martinez A.E., Ljungberg M.C., Lanza D.G., De Giorgi M., Chuecos M.A., Alves-Bezerra M. (2025). A comprehensive atlas of AAV tropism in the mouse. Mol. Ther..

[17] Schoenberger S.P., Toes R.E., van der Voort E.I., Offringa R., Melief C.J. (1998). T-cell help for cytotoxic T lymphocytes is mediated by CD40-CD40L interactions. Nature.

[18] Janssen E.M., Lemmens E.E., Wolfe T., Christen U., von Herrath M.G., Schoenberger S.P. (2003). CD4+ T cells are required for secondary expansion and memory in CD8+ T lymphocytes. Nature.

[19] Bedoui S., Heath W.R., Mueller S.N. (2016). CD4(+) T-cell help amplifies innate signals for primary CD8(+) T-cell immunity. Immunol. Rev..

[20] Wang Z.Y., Chen T.T., Liu W.X., Xu N.N., Xu Y.Q., Li N., Gao P.P., Wei W., Wu G.Y., Sun W.Y. (2025). β-arrestin2 depletion attenuates autoimmune hepatitis in mice via preventing intestinal barrier disruption and inhibiting ERS mediated intestinal epithelial cells apoptosis. Biochem. Pharmacol..

[21] Pujals A., Favre L., Pioche-Durieu C., Robert A., Meurice G., Le Gentil M., Chelouah S., Martin-Garcia N., Le Cam E., Guettier C. (2015). Constitutive autophagy contributes to resistance to TP53-mediated apoptosis in Epstein-Barr virus-positive latency III B-cell lymphoproliferations. Autophagy.

[22] Li X., Li F., Zhang X., Zhang H., Zhao Q., Li M., Wu X., Wang L., Liu J., Wu X. (2022). Caspase-8 auto-cleavage regulates programmed cell death and collaborates with RIPK3/MLKL to prevent lymphopenia. Cell Death Differ..

[23] Mognol G.P., de Araujo-Souza P.S., Robbs B.K., Teixeira L.K., Viola J.P. (2012). NFAT1-dependent regulation of the c-Myc promoter involves negative and positive NFAT-responsive elements. Cell Cycle.

[24] Kim S., Park C.I., Lee S., Choi H.R., Kim C.H. (2023). Reprogramming of IL-12 secretion in the PDCD1 locus improves the anti-tumor activity of NY-ESO-1 TCR-T cells. Front. Immunol..

[25] Macián F. (2005). NFAT proteins: key regulators of T-cell development and function. Nat. Rev. Immunol..

