# Supplementary material for: Activation-gated, T cell-restricted silencing of Dapk1 enhances Bacille Calmette-Guérin-elicited protective CD4+ memory
Source: iScience. 2026 Apr 3;29(5):115593. doi: 10.1016/j.isci.2026.115593 (PMC13125903; doi:10.1016/j.isci.2026.115593)
Supplement: Document S1. Figures S1–S5 [file mmc1.pdf]

iScience, Volume *29*

Supplemental information

Activation-gated, T cell-restricted silencing of *Dapk1* enhances Bacille Calmette-Guérin-elicited protective $CD4^+$ memory

Zixuan Liu, Weihuang Liu, Chenyu Zhao, Xuanchang Bai, Fangting Zhou, Hang Sun, Jiahe Shi, Pinru Chen, Min Liu, and Qin Pan

Fig. S1

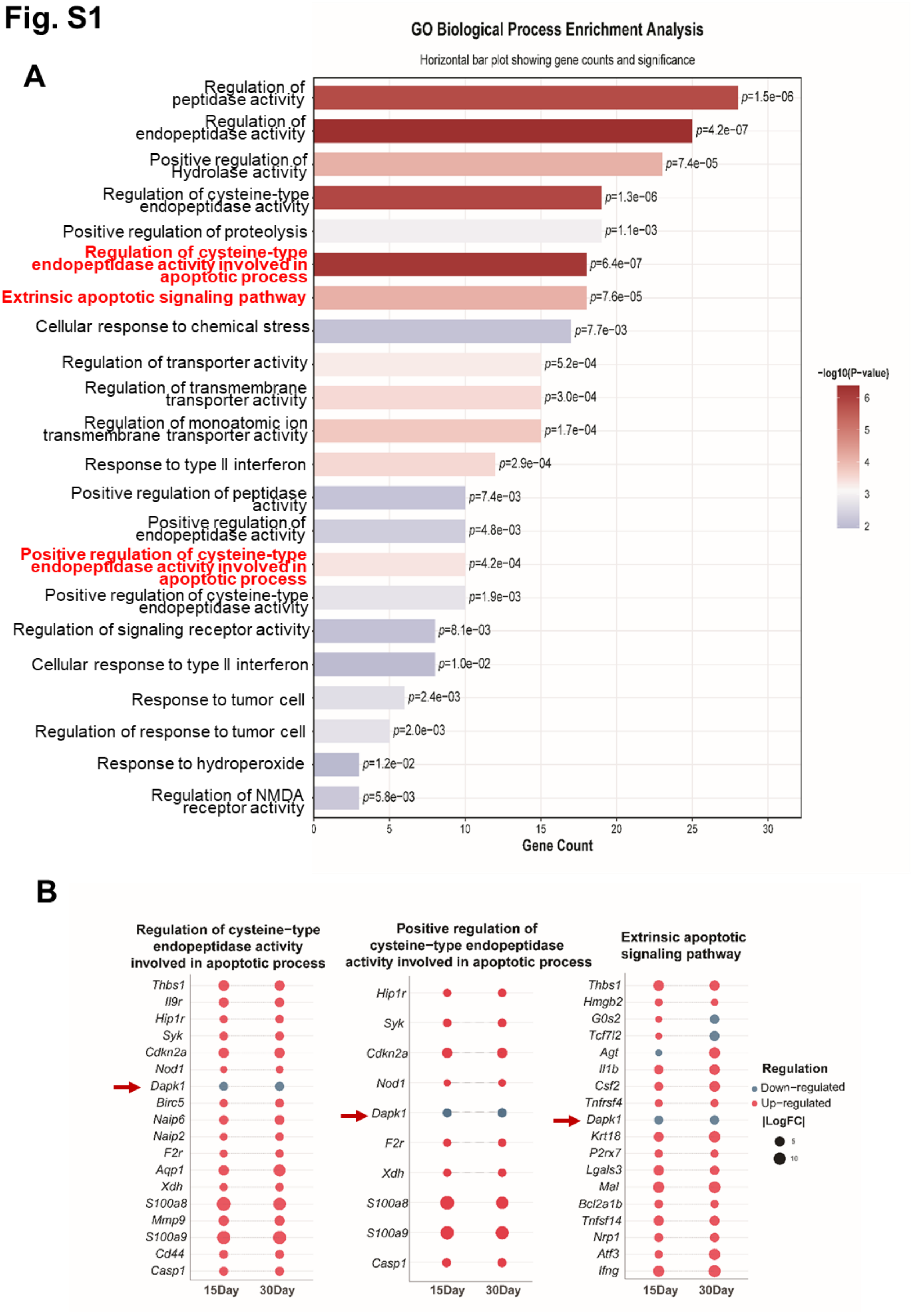
A
GO Biological Process Enrichment Analysis
Horizontal bar plot showing gene counts and significance
Regulation of peptidase activity
Regulation of endopeptidase activity
Positive regulation of Hydrolase activity
Regulation of cysteine-type endopeptidase activity
Positive regulation of proteolysis
Regulation of cysteine-type endopeptidase activity involved in apoptotic process
Extrinsic apoptotic signaling pathway
Cellular response to chemical stress
Regulation of transporter activity
Regulation of transmembrane transporter activity
Regulation of monoatomic ion transmembrane transporter activity
Response to type Ⅱ interferon
Positive regulation of peptidase activity
Positive regulation of endopeptidase activity
Positive regulation of cysteine-type endopeptidase activity involved in apoptotic process
Positive regulation of cysteine-type endopeptidase activity
Regulation of signaling receptor activity
Cellular response to type Ⅱ interferon
Response to tumor cell
Regulation of response to tumor cell
Response to hydroperoxide
Regulation of NMDA receptor activity
p=1.5e−06
p=4.2e−07
p=7.4e−05
p=1.3e−06
p=1.1e−03
p=6.4e−07
p=7.6e−05
p=7.7e−03
p=5.2e−04
p=3.0e−04
p=1.7e−04
p=2.9e−04
p=7.4e−03
p=4.8e−03
p=4.2e−04
p=1.9e−03
p=8.1e−03
p=1.0e−02
p=2.4e−03
p=2.0e−03
p=1.2e−02
p=5.8e−03
0 5 10 15 20 25 30
Gene Count
−log10(P−value)
6 5 4 3 2
B
Regulation of cysteine−type endopeptidase activity involved in apoptotic process
Thbs1
Il9r
Hip1r
Syk
Cdkn2a
Nod1
Dapk1
Birc5
Naip6
Naip2
F2r
Aqp1
Xdh
S100a8
Mmp9
S100a9
Cd44
Casp1
15Day 30Day
Positive regulation of cysteine−type endopeptidase activity involved in apoptotic process
Hip1r
Syk
Cdkn2a
Nod1
Dapk1
F2r
Xdh
S100a8
S100a9
Casp1
15Day 30Day
Extrinsic apoptotic signaling pathway
Thbs1
Hmgb2
G0s2
Tcf7l2
Agt
Il1b
Csf2
Tnfrsf4
Dapk1
Krt18
P2rx7
Lgals3
Mal
Bcl2a1b
Tnfsf14
Nrp1
Atf3
Ifng
15Day 30Day
Regulation
Down−regulated
Up−regulated
|LogFC|
5
10

Supplementary Figure S1 (related to Figure 1). Transcriptional profiling identifies apoptotic pathways enriched in CD44^{hi}CD4^{+} T cells. (A) Enrichment of cell death- and stress response-related biological processes. Gene Ontology (GO) enrichment analysis (Biological Process domain) of differentially expressed genes (DEGs) between CD44^{hi} and CD44^{lo} CD4^{+} T cells isolated 15 days and 30 days post-BCG vaccination. The bars display the twenty-two most significantly enriched GO terms (P<0.05) related to cell death and stress responses. (B) *Dapk1* is a common downregulated gene across three core apoptotic pathways. Bubble plots showing the $\log_2$ (fold change) of individual genes within three key apoptotic GO term sets: (i) extrinsic apoptotic signaling pathway, (ii) regulation of cysteine-type endopeptidase activity involved in apoptotic process, and (iii) positive regulation of cysteine-type endopeptidase activity involved in apoptotic process. Bubble size corresponds to the absolute value of $\log_2$FC.

Fig. S2

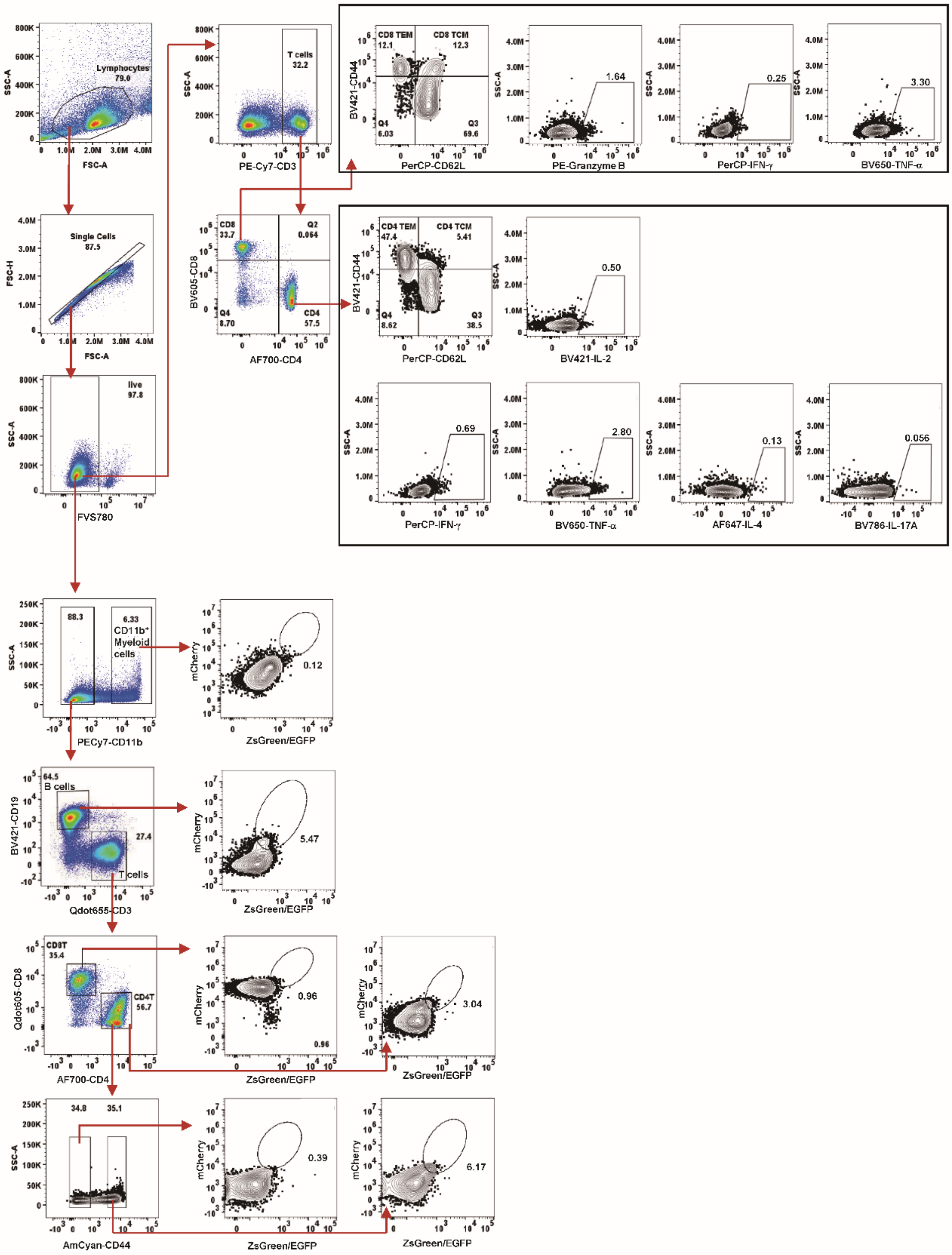

Supplementary Figure S2 (related to Figure 2). FCM gating for memory subsets and reporter detection.

Fig. S3

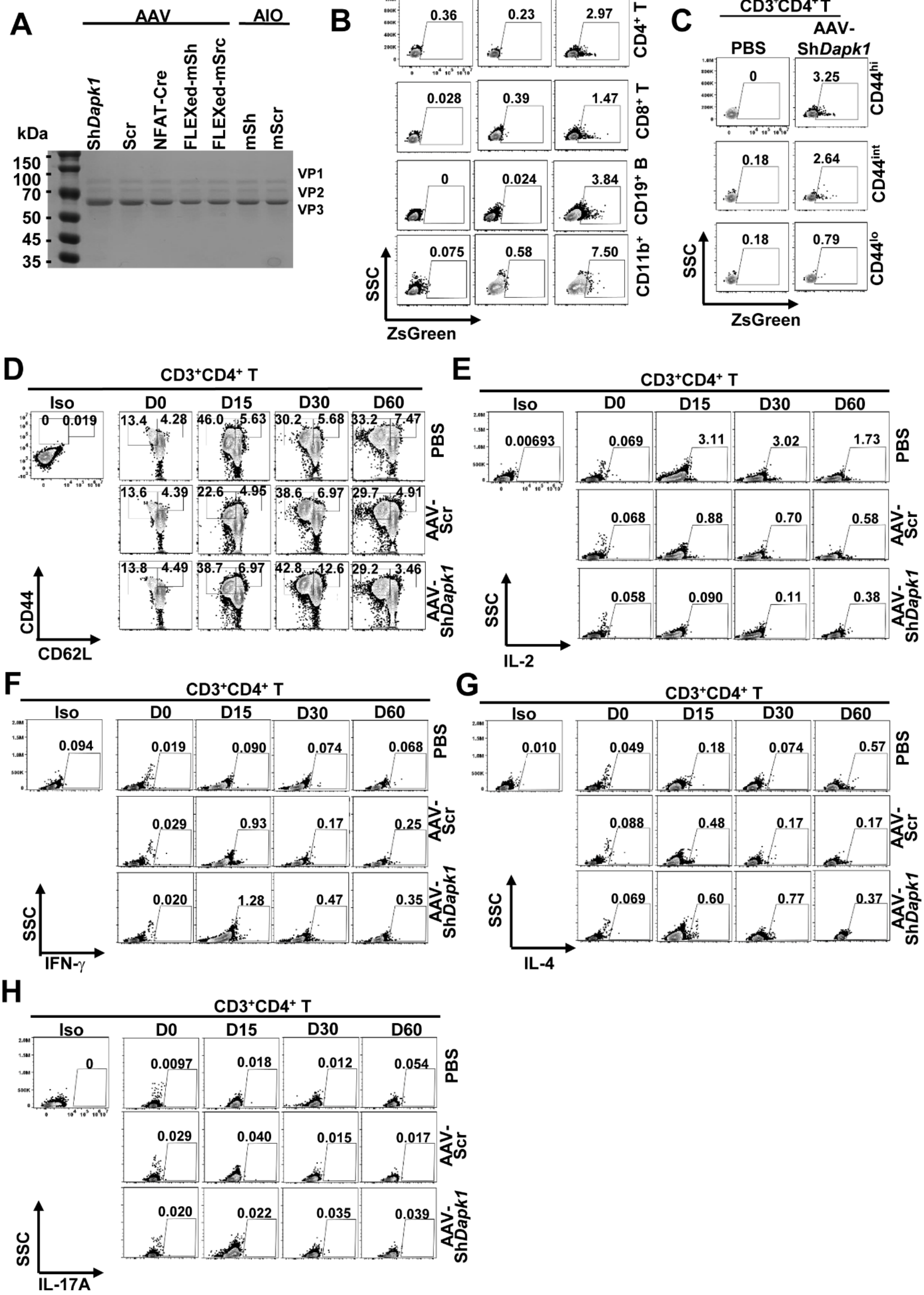
A
AAV
AIO
ShDapk1
Scr
NFAT-Cre
FLEXed-mSh
FLEXed-mSrc
mSh
mScr
kDa
150
100
70
50
45
35
VP1
VP2
VP3
B
Unstained
PBS
AAV-ShDapk1
0.36
0.23
2.97
CD4+ T
0.028
0.39
1.47
CD8+ T
0
0.024
3.84
CD19+ B
0.075
0.58
7.50
CD11b+
SSC
ZsGreen
C
CD3+CD4+ T
PBS
AAV-ShDapk1
0
3.25
CD44hi
0.18
2.64
CD44int
0.18
0.79
CD44lo
SSC
ZsGreen
D
CD3+CD4+ T
Iso
D0
D15
D30
D60
0
0.019
13.4
4.28
46.0
5.63
30.2
5.68
33.2
7.47
PBS
13.6
4.39
22.6
4.95
38.6
6.97
29.7
4.91
AAV-Scr
13.8
4.49
38.7
6.97
42.8
12.6
29.2
3.46
AAV-ShDapk1
CD44
CD62L
E
CD3+CD4+ T
Iso
D0
D15
D30
D60
0.00693
0.069
3.11
3.02
1.73
PBS
0.068
0.88
0.70
0.58
AAV-Scr
0.058
0.090
0.11
0.38
AAV-ShDapk1
SSC
IL-2
F
CD3+CD4+ T
Iso
D0
D15
D30
D60
0.094
0.019
0.090
0.074
0.068
PBS
0.029
0.93
0.17
0.25
AAV-Scr
0.020
1.28
0.47
0.35
AAV-ShDapk1
SSC
IFN-γ
G
CD3+CD4+ T
Iso
D0
D15
D30
D60
0.010
0.049
0.18
0.074
0.57
PBS
0.088
0.48
0.17
0.17
AAV-Scr
0.069
0.60
0.77
0.37
AAV-ShDapk1
SSC
IL-4
H
CD3+CD4+ T
Iso
D0
D15
D30
D60
0
0.0097
0.018
0.012
0.054
PBS
0.029
0.040
0.015
0.017
AAV-Scr
0.020
0.022
0.035
0.039
AAV-ShDapk1
SSC
IL-17A

Supplementary Figure S3 (related to Figure 2). AAV preparation and representative flow cytometry analysis of ZsGreen expression and cytokine production in the mice treated with AAV-ZsGreen-sh*Dapk1*

(A) AAVs were prepared as described in the Methods and viral capsid proteins were analyzed by SDS-PAGE.

(B)-(C) Mice were intravenously administered AAV2/8-ZsGreen-sh*Dapk1* on day 0. ZsGreen expression was measured by FCM. Representative flow plots are shown. (B) ZsGreen expression in splenic $CD4^+$ T, $CD8^+$ T, $CD19^+$ B and $CD11b^+$ myeloid cells. (C) ZsGreen expression in splenic $CD44^{hi}CD4^+$, $CD44^{int}CD4^+$ and $CD44^{lo}CD4^+$T cells.

(D)-(H) Analysis of memory and cytokine-producing $CD4^+$ T cells following BCG immunization. On day -5, mice were intravenously injected with AAV2/8-ZsGreen-sh*Dapk1*. On day 0, these mice were immunized intravenously with BCG. Representative flow plots are shown.

(D) Longitudinal frequencies of T_{CM} and T_{EM} $CD4^+$ T cells.

(E)-(H) Cytokine production was measured by FCM following ex vivo stimulation with inactivated BCG.

Fig. S4

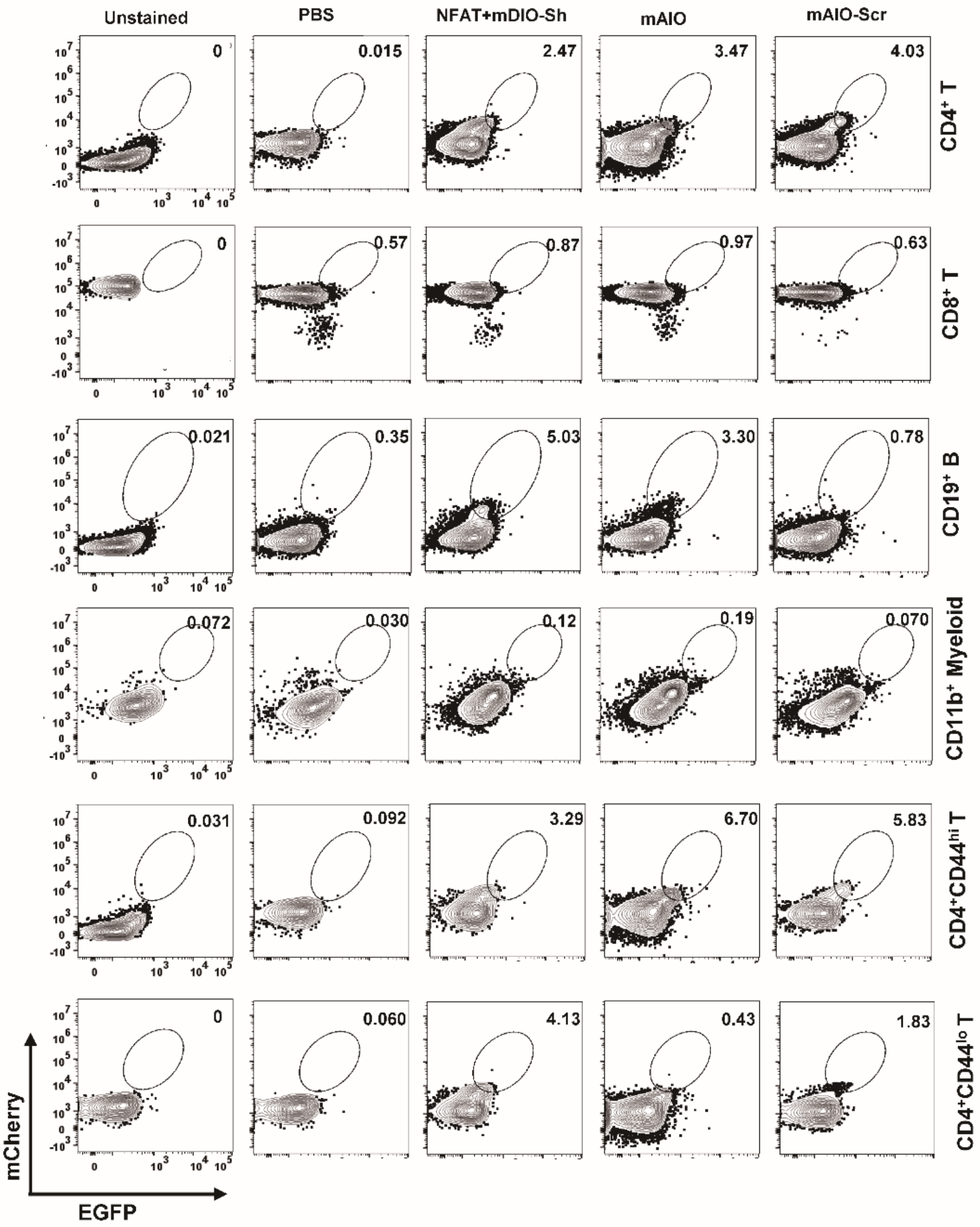

Supplementary Figure S4 (related to Figure 6). FCM analysis of in vivo lineage distribution and double-reporter mapping in the mice treated with AAV-AIO-mSh or NFAT/FLEXed-mSh. Mice were administrated with AAVs encoding a *Dapk1*-targeting shRNA driven by either AIO-mSh or NFAT/FLEXed-mSh. The distribution of EGFP^{+}/mCherry^{+} double-positive cells across lineages was measured 20 days after administration. Representative flow cytometry plots are shown.

Fig. S5

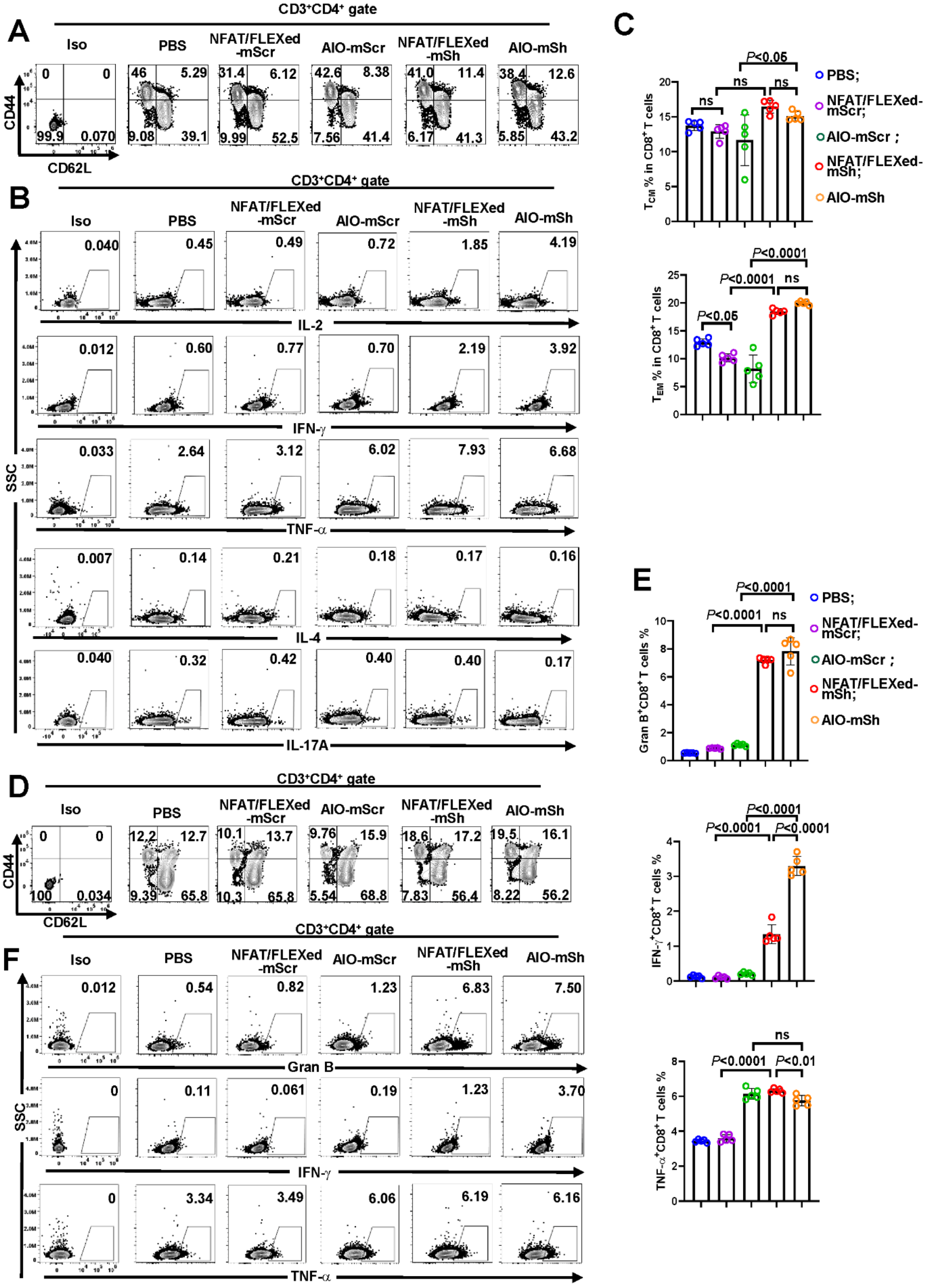
A
CD3⁺CD4⁺ gate
Iso
PBS
NFAT/FLEXed -mScr
AIO-mScr
NFAT/FLEXed -mSh
AIO-mSh
CD44
CD62L
0 0 99.9 0.070
46 5.29 9.08 39.1
31.4 6.12 9.99 52.5
42.6 8.38 7.56 41.4
41.0 11.4 6.17 41.3
38.4 12.6 5.85 43.2
B
CD3⁺CD4⁺ gate
SSC
IL-2
0.040 0.45 0.49 0.72 1.85 4.19
IFN-γ
0.012 0.60 0.77 0.70 2.19 3.92
TNF-α
0.033 2.64 3.12 6.02 7.93 6.68
IL-4
0.007 0.14 0.21 0.18 0.17 0.16
IL-17A
0.040 0.32 0.42 0.40 0.40 0.17
C
TCM % in CD8⁺ T cells
TEM % in CD8⁺ T cells
P<0.05
ns
P<0.0001
PBS;
NFAT/FLEXed-mScr;
AIO-mScr ;
NFAT/FLEXed-mSh;
AIO-mSh
D
CD3⁺CD4⁺ gate
0 0 100 0.034
12.2 12.7 9.39 65.8
10.1 13.7 10.3 65.8
9.76 15.9 5.54 68.8
18.6 17.2 7.83 56.4
19.5 16.1 8.22 56.2
E
Gran B⁺CD8⁺ T cells %
IFN-γ⁺CD8⁺ T cells %
TNF-α⁺CD8⁺ T cells %
P<0.0001
P<0.01
F
CD3⁺CD4⁺ gate
Gran B
0.012 0.54 0.82 1.23 6.83 7.50
IFN-γ
0 0.11 0.061 0.19 1.23 3.70
TNF-α
0 3.34 3.49 6.06 6.19 6.16

Supplementary Figure S5(related to Figure 7). FCM analysis of memory T cells and cytokine production in the murine challenge model.

(A)-(F) Mice were administered AAVs encoding a *Dapk1*-targeting shRNA under the control of either AIO-mSh or NFAT/FLEXed-mSh 5 days before BCG immunization. Intranasal infection with *M.tb* H37Ra was performed 30 days post-immunization.

(A) FCM analysis of memory $CD4^+$ T cells 30 days post BCG immunization. Representative flow plots.

(B) FCM analysis of cytokine-producing $CD4^+$ T cells after ex vivo restimulation with BCG. Representative flow plots.

(C)-(D) FCM analysis of memory $CD8^+$ T cells 30 days post BCG immunization. (C) pooled data (n=5). (D) Representative flow plots.

(E)–(F) FCM analysis of cytokine-producing $CD8^+$ T cells after ex vivo restimulation with BCG. (E) pooled data (n=5). (F) Representative flow plots.

Data are presented as the mean ± SD. Statistical significance was assessed using one-way ANOVA followed by Tukey's post hoc test.
